# Evolution, Expression Differentiation and Interaction Specificity of Heterotrimeric G-Protein Subunit Gene Family in the Mesohexaploid *Brassica rapa*

**DOI:** 10.1371/journal.pone.0105771

**Published:** 2014-09-05

**Authors:** Gulab C. Arya, Roshan Kumar, Naveen C. Bisht

**Affiliations:** National Institute of Plant Genome Research, New Delhi, India; National Institute of Plant Genome Research, India

## Abstract

Heterotrimeric G-proteins, comprising of Gα, Gβ, and Gγ subunits, are important signal transducers which regulate many aspects of fundamental growth and developmental processes in all eukaryotes. Initial studies in model plants *Arabidopsis* and rice suggest that the repertoire of plant G-protein is much simpler than that observed in metazoans. In order to assess the consequence of whole genome triplication events within Brassicaceae family, we investigated the multiplicity of G-protein subunit genes in mesohexaploid *Brassica rapa*, a globally important vegetable and oilseed crop. We identified one Gα (*BraA.Gα1*), three Gβ (*BraA.Gβ1, BraA.Gβ2*, and *BraA.Gβ3*), and five Gγ (*BraA.Gγ1, BraA.Gγ2, BraA.Gγ3, BraA.Gγ4*, and *BraA.Gγ5*) genes from *B. rapa*, with a possibility of 15 Gαβγ heterotrimer combinations. Our analysis suggested that the process of genome triplication coupled with gene-loss (gene-fractionation) phenomenon have shaped the quantitative and sequence diversity of G-protein subunit genes in the extant *B. rapa* genome. Detailed expression analysis using qRT-PCR assays revealed that the G-protein genes have retained ubiquitous but distinct expression profiles across plant development. The expression of multiple G-protein genes was differentially regulated during seed-maturation and germination stages, and in response to various phytohormone treatments and stress conditions. Yeast-based interaction analysis showed that G-protein subunits interacted in most of the possible combinations, with some degree of subunit-specific interaction specificity, to control the functional selectivity of G-protein heterotrimer in different cell and tissue-types or in response to different environmental conditions. Taken together, this research identifies a highly diverse G-protein signaling network known to date from *B. rapa*, and provides a clue about the possible complexity of G-protein signaling networks present across globally important *Brassica* species.

## Introduction

Heterotrimeric G-proteins (hereafter G-proteins) are a class of signal transduction proteins that provide a key mechanism by which extracellular signals are transmitted to cell milieu, across phyla. The three distinct subunits namely, Gα (G-alpha), Gβ (G-beta), and Gγ (G-gamma) along with GPCRs (G-protein coupled receptors) are the fundamental components of G-protein signaling complex, wherein, the Gα subunit acts as a switch and found in either GDP bound ‘OFF’ or in GTP bound ‘ON’ form [1]. Signal perception through GPCR causes change in the conformation of Gα subunit leading to exchange of GTP for GDP (i.e. GTP binding). The exchange of GTP molecule leads to the dissociation of heterotrimer into Gα-GTP and Gβγ dimer; with both components freely interacting with their downstream ‘effectors’. Intrinsic GTPase (i.e. GTP hydrolyzing) activity of Gα causes conversion of GTP bound ‘ON’ form of Gα to GDP bound ‘OFF’ form, leading to re-association of heterotrimer and inhibition of signal perception from the cell surface [1].

The core G-protein components have structural similarities and are evolutionary conserved across phyla. However, among higher organisms, particularly the plant and animal models seem to have significant quantitative and regulatory differences in G-protein signaling [2]. For example, the G-protein components present in plants are strikingly simpler than those observed in metazoans. The human genome encodes well-characterized 23 Gα, five Gβ and 12 Gγ proteins and >800 GPCR for performing multiple diverse cellular processes, whereas the model plants *Arabidopsis* and rice have only one canonical Gα and Gβ proteins and 3-5 Gγ proteins [2], [3]. Till date, the most elaborate and diverse G-proteins family reported from polyploid soybean genome encodes four Gα, four Gβ and ten Gγ proteins [4], [5]. Biochemical evidences further suggested that the plant kingdom uses a distinct regulatory system in G-protein signaling. The plant Gα proteins showed ‘self-activating’ property, having significantly higher GTP-binding rates and a slower GTPase activity, which is in contrast to the mammalian Gα proteins, thereby indicating that the plant G-proteins remain mostly in constitutively active state [6], [7].

Although quantitatively simpler, analysis of G-protein functions in plants has emphasized the vital functions they play in plant growth and development, and in response to various environmental cues [2]. G-protein regulates various biological processes like seed germination [8], [9], stomatal opening and closing [10], [11], leaf shape and size, silique shape and size, flower development [12], root development [13] and cell growth and elongation [14]. G-proteins are also involved in several signaling pathways, including hormones signaling [9], [15]–[17], sugar perception [8], [13], [16], blue and red light mediated responses [18], [19] and pathogen resistance [20], [21]. Furthermore, G-proteins also regulate key agronomical traits in crops such as regulation of transpiration efficiency [22], dwarfism [15], [17], panicle morphology, seed size and yield [23], [24], stress tolerance [25] and oil production [26] as well as nodulation in legumes [27].

The genus *Brassica* is one of the most important genus within Brassicaceae (Cruciferae or mustard family) and includes many species that are grown as crops having important roles in global agriculture, horticulture, and human health. Within this genus, six species are cultivated globally: *Brassica rapa* (AA genome; 2n = 20), *B. nigra* (BB; 2n = 16), *B. oleracea* (CC; 2n = 18), *B. juncea* (AABB; 2n = 36), *B. napus* (AACC; 2n = 38), and *B. carinata* (BBCC; 2n = 34). Their genomic relationships are well characterized, wherein the latter three species were derived from natural hybridization and polyploidization of two of the former three diploid taxa [28]. The cultivated *Brassica* crops have been investigated at morphological, biochemical, and genetic level for decades, which led to the identification of genomic regions (i.e. quantitative trait loci or QTL) that control key agronomical traits in *Brassica* species. However, comprehensive data from a *Brassica* species on the important roles played by G-proteins in regulating fundamental growth and development processes is largely limiting at present.

Among the six *Brassica* species, *B. rapa* cultivars are globally used as leafy vegetables, vegetable oils, turnip roots, turnip greens, turnip tops, and fodder turnip. With the availability of genetic and genomic resources in recent years, *B. rapa* has also emerged as a potential model crop of the *Brassica* genus [29]. Nonetheless, it is one of the key progenitor species which contributes A-genome to the globally important allopolyploid crops, *B. juncea* and *B. napus* [30]. The, yet simpler, genome of *B. rapa* is considered to be mesohexaploid, evolved by whole genome-triplication events (1R, 2R, 3R, in order) from a common ancestor shared with the model plant *A. thaliana* [31], [32]. Sequence level information in recent years have confirmed that *B. rapa* contains three sub-genomes namely, least fractionized (LF), moderately gene fractionized (MF1) and most gene fractionized (MF2) sub-genomes; and these three sub-genomes further encompass 1-6 copies of 24 distinct genomic blocks (A-X) of ancestral Brassicaceae karyotype [29]–[33]. Interestingly, genome-wide comparative analysis of the *B. rapa* gene space has revealed genome shrinkage and differential loss of duplicated genes after whole genome-triplication [31]. As a consequence, up to three copies of orthologous genomic regions of *A. thaliana* can be observed in the extant diploid *Brassica* genomes [32], thereby providing sub or neo-functionalization of duplicated genes.

The availability of sequence assembly of the *B. rapa* genome (http://www.brassicadb.org/) [29] led us to investigate the consequence of genome-triplication events towards shaping the evolution and complexity of G-protein signaling network present within Brassicaceae family. This study describes the isolation of multiple G-protein subunit genes from the mesohexaploid *B. rapa* genome; compares their gene-sequence and structural diversity; details their expression profiles across plant development, including both vegetative and reproductive stages and responses under various environmental stresses; and evaluates the specificity of interactions among multiple G-protein subunits. The identification and molecular characterization of an elaborate G-protein repertoire from *B. rapa* supports the presence of highly diverse G-protein signaling networks across polyploid *Brassica* species.

## Materials and Methods

### Plant materials and growth conditions

*B. rapa* L. cv. YID1 was grown under controlled growth conditions at day (24°C, 10 hrs, 300 µmol m^−2^ s^−1^) and night (18°C, 14 hrs) photoperiod with 60% relative humidity. Different developmental tissues consisting of vegetative (seedling, shoot apex, stem, root, and leaf) and reproductive stages (flower, silique 3 days post anthesis (dpa), 7 dpa, 14 dpa, 21 dpa, 28 dpa, and 35 dpa) were collected and frozen immediately in liquid nitrogen and stored at −80°C. For germination assays, seeds were grown on germination paper and different stages were collected at an interval of 3, 6, 12 and 24 hrs after seed-imbibitions, snap frozen in liquid nitrogen, and stored at −80°C.

For stress treatment experiments, five days old seedlings were used. The treatments performed in the current study were described previously [34]. Briefly, surface sterilized seeds (using 0.05% mercuric chloride and 1% sodium hypochlorite) were grown on half strength MS media and then transferred to liquid media for 24 hrs in dark. Thereafter, seedlings were transferred to a conical flask containing abscisic acid (ABA, 100 µM), sodium chloride (NaCl, 300 µM), indole acetic acid (IAA, 100 µM), salicylic acid (SA, 200 µM) and methyl jasmonic acid (MeJa, 300 µM) for 3 and 6 hrs each. For cold and heat treatments, seedlings were placed at 4°C and 42°C respectively. Seedlings without treatments were used as control. Samples were collected, frozen in liquid nitrogen, and stored at −80°C.

### Cloning of *B. rapa* G-protein genes

The G-protein genes of *B. rapa* were identified by performing BLAST (blastn and tblastx) analysis on the updated *B. rapa* genome assembly available at BRAD database v1.2 (http://www.brassicadb.org/), with *Arabidopsis* (At2g26300, At4g34460, At3g63420, At3g22942, At5g20635) and rice (Os05g26890, Os03g46650, Os03g43480, Os02g04520) full-length genes as queries. From the significant hits showing lower E-value, sequences containing full length or nearly full length reading frames were identified. Full length coding DNA sequences of G-protein subunit genes were amplified from cDNA representing various developmental stages of *B. rapa* cv. YID1 using gene-specific primers (Table S1). The G-protein subunit genes amplified from *B. rapa* genome were cloned into pENTR/D-TOPO vector and further confirmed by sequencing.

### RNA isolation and quantitative real time PCR

Total RNA was isolated from different plant tissues using Spectrum Plant Total RNA kit (Sigma Aldrich, USA). The purity and quality of the isolated RNA was determined by OD260:280 nm absorption ratio which were found to be in range of (1.8–2.0) for all samples and the integrity of the isolated RNA was determined by running them on 1.2% agarose gel containing formaldehyde. Approximately two microgram of total RNA was used to synthesis cDNA using High Capacity cDNA Reverse Transcription kit (Applied Biosystems, USA). The cDNAs, diluted to 1∶50, were used as template for quantitative real time PCR analysis using gene specific primers (Table S1) in ABI7900-HT fast real time PCR machine (Applied Biosystems, USA). *Actin* and *GAPDH* genes of *Brassica* origin were used as endogenous controls [34]. The data represents mean of at least three independent biological samples, with two technical replicates each. Statistical analysis were conducted using one-way ANNOVA following Fishers LSD test of significance at 95% confidence value (p<0.05).

### Protein-protein interaction assays

The interaction between Gα and Gβ subunit proteins was performed using the mating-based split ubiquitin system [35]. Full length CDS of Gα was cloned into vectors containing the N-terminal half of ubiquitin (both wild-type (Nub-wt) and a low-affinity NubG version; Trp+) whereas the full length CDS of Gβ subunit genes were cloned into vector containing the C-terminal half of ubiquitin (Cub; Leu+). Gα and Gβ vectors were transformed into yeast haploid strains THY.AP5 (MATα) and THY.AP4 (MATa), respectively. Mating of haploid cells was performed subsequently and the strength of interaction was determined by the growth of diploid yeast colonies on minimal media (synthetic dextrose) lacking adenine, histidine, leucine and tryptophan (-AHLT), but containing variable concentrations of methionine at 0, 250 and 500 µM.

To determine the interaction between Gβ and Gγ subunit proteins, GATEWAY-based yeast-two-hybrid assay was performed (ProQuest Yeast Two Hybrid System, Invitrogen, Life Technologies, USA). Briefly, CDS of Gβ subunits were cloned into pDEST32 bait vector (containing DNA-binding domain) and Gγ subunits were cloned into pDEST22 prey vector (containing DNA-activating domain). Both vectors were co-transformed into yeast host strain MaV203 as per the manufacturer's instructions. The quantitative interaction of proteins was quantified by β-galatosidase (β-gal) expression assay using o-nitrophenyl-β-D-galactopyranoside (ONPG) as a substrate and also determined by growth of the diploid yeast colonies on minimal media lacking His, Leu and Trp but containing 0 mM, 25 mM, and 100 mM of 3AT (3-Amino-1,2,4-triazole). At least three independent biological experiments were performed.

### Phylogenetic and divergence analysis

Homologs of Gα, Gβ and Gγ subunit proteins from various plant species were identified using the BLAST search analysis on genome databases available at Phytozome (http://www.phytozome.net/) and other publicly available databases. Multiple sequence alignment of the full length proteins sequences of representative Gα, Gβ and Gγ proteins was performed by ClustalW and the evolutionary tree was constructed using the Neighbor-Joining method in MEGA5 [36], adopting the pairwise deletion option of the gaps, and the Poisson substitution method with 1000 replicated bootstrap values. Following plant species were used for phylogenetic tree construction: *Arabidopsis thaliana (At), Oryza sativa (Os), Glycine max (Glyma), Brassica napus (Bna), Zea mays (GRMZM), Medicago trunculata (Medtr)* and *Psycomitrella patens (Pp)*.

To determine the divergence time coding DNA sequences of *B. rapa* G-protein genes were aligned with the orthologues sequences from *Arabidopsis* using ClustalW. Synonymous (Ks) and non-synonymous (Ka) base substitution were calculated by DnaSP v5 [37]. The divergence time for G-protein genes was calculated from Ks value using the equation T = Ks/(2x[1.5x10^−8^] where 1.5 x 10^−8^ substitution per site per year is the synonymous mutation rate reported for *Brassica* genus [38].

### Accession numbers

The coding DNA sequences (CDS) isolated in the current study were submitted to GenBank with accession nos. KJ451019 (*BraA.Gα1* CDS); KJ451021 (*BraA.Gβ1* CDS); KJ451023 (*BraA.Gβ2* CDS); KJ451025 (*BraA.Gβ3* CDS); KJ451027 (*BraA.Gγ1* CDS); KJ451029 (*BraA.Gγ2* CDS); KJ451031 (*BraA.Gγ3* CDS); KJ451033 (*BraA.Gγ4* CDS) and KJ451035 (*BraA.Gγ5* CDS).

## Results

### Identification and sequence analysis of genes encoding heterotrimeric G-protein subunits from *B. rapa*

*B. rapa* is a mesohexaploid crop with three sub-genomes, sharing the same diploid ancestor with the model plant species *A. thaliana* [32], [33]. To identify the inventories of core G-protein genes from *B. rapa*, we queried the BRAD database (v1.2), which provides the draft genome of recently sequenced *B. rapa* (cv. Chiifu-401) [29]. BLAST analysis of the *Arabidopsis* and rice G-protein sequences identified a total of one Gα, three Gβ and five Gγ subunits encoding genes in *B. rapa* genome (Table 1). Based on sequence information, full-length coding DNA sequences (CDS) were isolated for each of the nine G-protein subunit genes using *B. rapa* cDNA and gene-specific primers; and confirmed with multiple amplifications from different tissue types. We named these sequences as Gα (*BraA.Gα1*), Gβ (*BraA.Gβ1, BraA.Gβ2*, and *BraA.Gβ3*), and Gγ (*BraA.Gγ1, BraA.Gγ2, BraA.Gγ3, BraA.Gγ4*, and *BraA.Gγ5*), following the universal nomenclature adopted for *Brassica* genes [39].

**Table 1:** Summary of G-protein genes isolated from *B. rapa*.

*B. rapa* gene	*B. rapa* gene ID#	Linkage group#	Size of full-length gene (bp)	Size of CDS (bp)	No. of exons	No. of intron	Size of protein (aa)
*BraA.Gα1*	Bra007761	A09	2420	1152	13	12	383
*BraA.Gβ1*	Bra017658	A03	1890	1137	06	05	378
*BraA.Gβ2*	Bra034628	A08	1934	1137	06	05	378
*BraA.Gβ3*	Bra011536	A01	2079	1134	06	05	377
*BraA.Gγ1*	Bra007741	A09	665	270	04	03	89
*BraA.Gγ2*	Bra001894	A03	805	306	04	03	101
*BraA.Gγ3*	Bra023782	A01	939	303	04	03	100
*BraA.Gγ4*	Bra020117	A02	2537	798	05	04	265
*BraA.Gγ5*	Bra006568	A03	4554	705	05	04	234

# - obtained from BRAD database (v1.2) (http://www.brassicadb.org/); bp - base pair; aa – amino acid.

**Figure 1:**
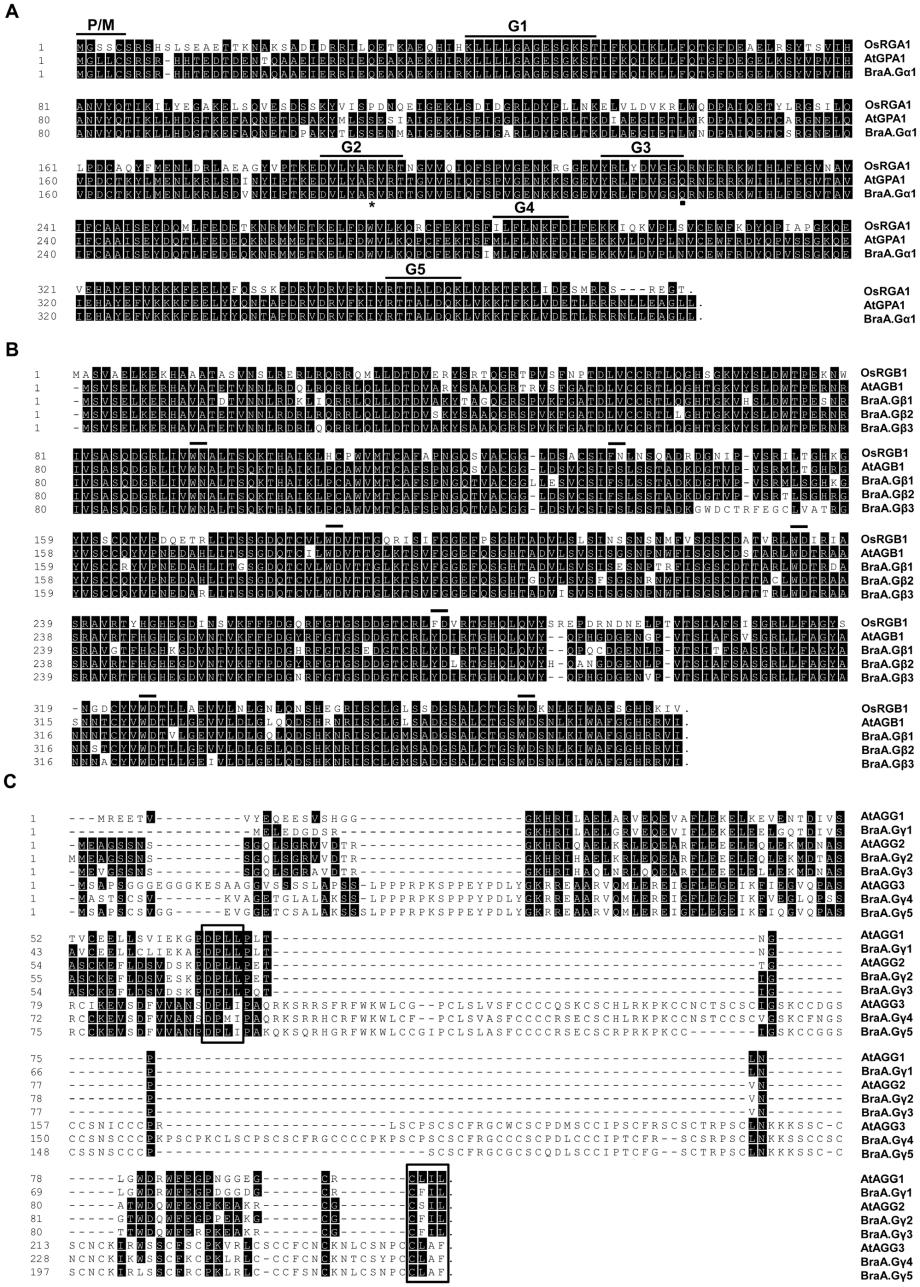
Amino acid sequence alignment of G-protein subunits of *B. rapa*. (**A**) The sequence alignment of *B. rapa* (BraA.Gα1) proteins with Gα protein of rice (OsRGA1) and *Arabidopsis* (AtGPA1). Consensus sequences for GTP binding and hydrolysis are labeled with G1–G5 boxes. P/M, the predicted site for palmitoylation/myritoylation (MGXXCS); closed square, the conserved glutamine (Q222) important for the GTPase activity of Gα proteins; *, the conserved ADP ribosylation site in Gα proteins. (**B**) Amino acid sequence alignment *B. rapa* (BraA.Gβ1-3) proteins with Gβ proteins of rice (OsRGB1) and *Arabidopsis* (AtAGB1) and. Seven WD repeats are marked with horizontal bar. (**C**) Amino acid sequence alignment of *B. rapa* (BraGγ1-5) proteins with *Arabidopsis* (AtAGG1-3) proteins. Conserved DPLL motif (provides hydrophobic contact to Gβ subunit proteins), and CaaX domain (required for attachment to cell-membrane) are marked within boxes, in order. The amino-acid sequence alignment of G-protein subunits was performed using ClustalW. The conserved and divergent residues are shaded in dark and white backgrounds, respectively.

Amino acid sequence alignment of deduced BraA.Gα1 protein with *Arabidopsis* Gα (AtGPA1) and rice Gα (OsRGA1) proteins showed 96.9% and 73.5% sequence identity, respectively (Figure 1A; Table S2). The deduced amino acid sequence of BraA.Gα1 protein contains conserved sequence for myristolyation and palmitoylation (MGXXCS) at its N-terminal end. Signature motifs for GTP binding and hydrolysis, designated as G1, G2, G3, G4 and G5 domains in Gα proteins were found to be conserved (Figure 1A). The putative ADP ribosylation target for cholera toxin and an invariant glutamine residue at position (Q222), which was previously used to generate a constitutively active form of Gα in both *Arabidopsis* and rice, were also conserved [40], [41]. The important lipid modification sites necessary for binding to Gβγ dimer were also present in BraA.Gα1 protein [41].

The *BraA.Gα1* gene sequence is mis-annotated in the current version of *B. rapa* genome assembly, predicting an ORF of 1143 bp, compared to 1152 bp full-length gene isolated in our study (Table 1). Our experimental data suggest that the predicted BraA.Gα1 protein showed deletion and perturbation of the G1 domain (Figure S1). The predicted cDNA could never be amplified in our experiments, even when using different primer combinations, various tissue types and across different cultivars of *B. rapa* including that of Chiffu-401, the sequenced cultivar (data not shown).

The deduced BraGβ proteins were highly homologous sharing 89.9–92.9% amino acid identity with *Arabidopsis* Gβ (AtAGB1), and 73.0–75.0% sequence identity with rice Gβ (OsRGB1) protein (Table S2). The BraGβ proteins contain seven WD (Trp-Asp) repeats critical for seven-bladed β-propeller structure of canonical Gβ protein (Figure 1B). Besides, the predicted coiled-coil N-terminal hydrophobic domain required for Gγ interaction and the sequences necessary for contact with Gα protein were also found to be conserved in BraGβ proteins [41].

Amino acid sequence alignment of deduced BraGγ proteins with *Arabidopsis* Gγ proteins (AtAGG1, AtAGG2, and AtAGG3) showed that BraGγ proteins were highly divergent sequences, sharing only 31.3–92.1% identity with *Arabidopsis* Gγ proteins and 30.0–86.3% of sequence identity among themselves (Table S2). Sequence comparison further revealed that the BraA.Gγ1 protein was the closest homolog of AtAGG1, whereas *B. rapa* genome encodes two homologs each of AtAGG2 (BraA.Gγ2 and BraA.Gγ3), and AtAGG3 (BraA.Gγ4 and BraA.Gγ5) (Table S2). All five BraGγ proteins consisted of DPLL motif, which is conserved across plant species and serves as important hydrophobic contact to Gβ subunit proteins [41]. In addition, an important signature sequence for isoprenyl modification, the CaaX box, required for its attachment to cell-membrane was also present at the C-terminus of all the five BraGγ subunit proteins (Figure 1C). Based on their C-terminal sequences, three proteins namely BraA.Gγ1, BraA.Gγ2 and BraA.Gγ3 could be assigned as plant type-A Gγ proteins, whereas BraA.Gγ4 and BraA.Gγ5 belonged to type-C Gγ proteins [3]. An extended plant specific cystein rich domain at C terminal was present in both BraA.Gγ4 and BraA.Gγ5 proteins, which is unique to only type-C Gγ proteins. No type-B Gγ proteins could be identified from *B. rapa* genome.

Thus with the isolation of nine G-protein gene sequences, a total of 15 Gαβγ heterotrimeric combinations are possible in mesohexaploid *B. rapa*, compared to only three heterotrimeric combinations reported in *Arabidopsis*, thereby making it the most elaborate and diverse G-protein signaling network known to date within Brassicaceae family.

### Phylogenetic relationship and divergence of *B. rapa* G-protein subunits

**Figure 2:**
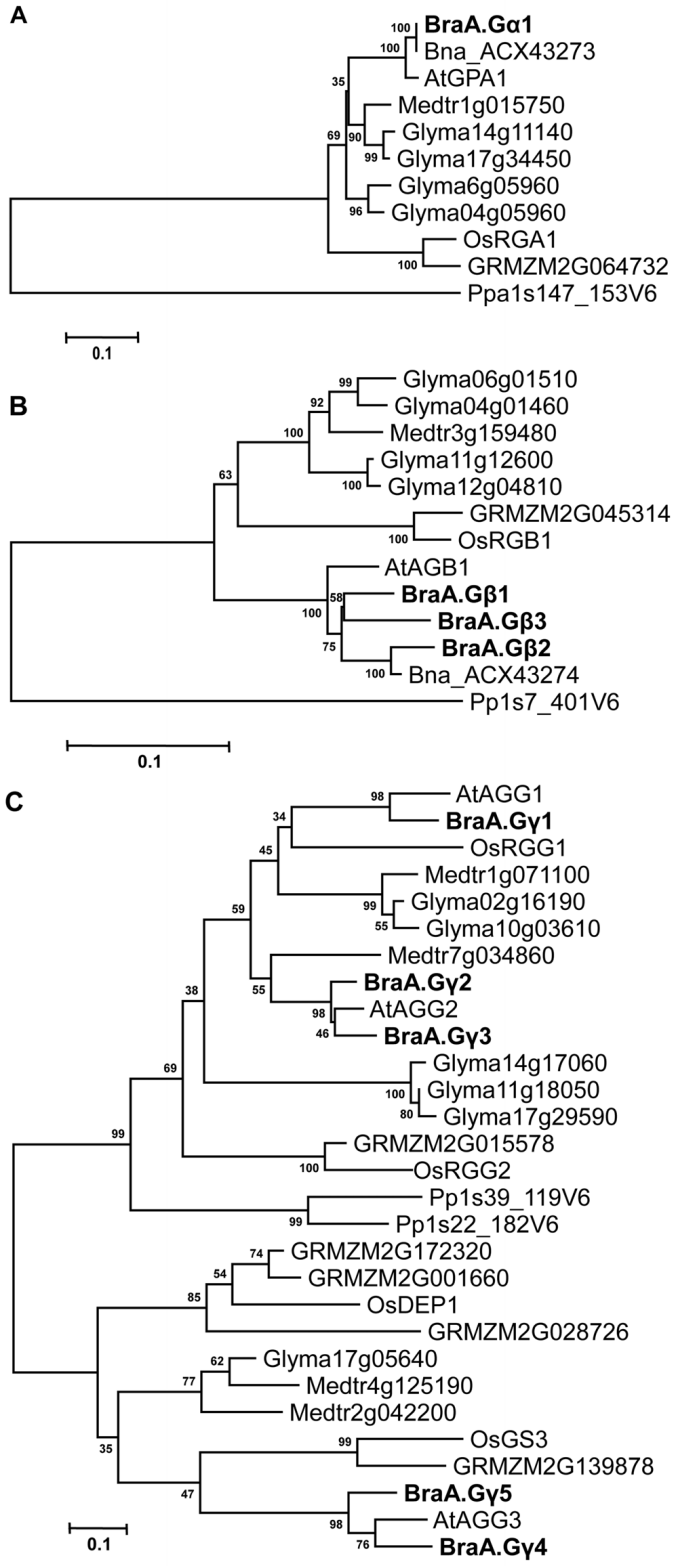
Phylogenetic relationship of G-protein subunits of *B. rapa*. The phylogenetic analysis of (**A**) Gα, (**B**) Gβ, and (**C**) Gγ proteins of *B. rapa* (Bra), *A. thaliana* (At), *O. sativa* (Os), *Glycine max* (Glyma), *Brassica napus* (Bna), *Zea mays* (GRMZM), *Medicago trunculata* (Medtr) and *Psycomitrella patens* (Pp) was performed using neighbor-joining method in MEGA5 (Tamura *et al.*, 2011). The values above the branches represent bootstrap percentage (1,000 replicates) of replicate trees in which the associated proteins clustered together. The tree is drawn to scale, with branch lengths measured in the number of substitutions per site. The *B. rapa* G-proteins are represented in bold letters.

A high level of amino acid identity and domain conservation indicated that the *B. rapa* G-proteins are closely related. The evolutionary relationship of *B. rapa* G-protein subunits were compared with G-protein sequences reported from other plant species (http://www.phytozome.net). On a neighbour-joining tree, the BraA.Gα1 protein grouped together with the Gα proteins reported from *B. napus* and the *Arabidopsis* AtGPA1 into a distinct Brassicaceae specific sub-group with high bootstrap support; whereas the Gα proteins reported from legume species and those from monocot lineages occupied separate subgroups (Figure 2A). Similarly, the Gβ proteins of Brassicaceae family branched into a group distinct from legumes, monocots, and lower plants (Figure 2B). The phylogenetic analysis clearly revealed that *B. rapa* has acquired additional copies of BraGβ proteins following triplication of its genome.

Phylogenetic analysis of Gγ proteins showed that the type-A BraGγ proteins (BraA.Gγ1–3) had evolved separately from the type-C BraGγ proteins (BraA.Gγ4 and BraA.Gγ5) (Figure 2C). The type-C BraGγ proteins grouped together with recently reported *Arabidopsis* AtAGG3 and two agronomically important proteins of rice, OsGS3 and OsDEP1 [2], [23], [24]. Interestingly, phylogenetic analysis revealed that AtAGG2 and BraA.Gγ3 (which form likely a pair of orthologs) was basal to BraA.Gγ2; whereas AtAGG3 and BraA.Gγ4 (again, a pair of orthologs) was basal to BraA.Gγ5. These observations suggest that although the BraGγ duplicated gene homologs share a close phylogeny with their corresponding *Arabidopsis* AGG2 and AGG3 counterparts, these might have diverge out significantly during the *Brassica* evolution.

We therefore estimated the divergence of G-protein subunit genes retained in extant *B. rapa* genome. The synonymous base substitution (Ks) value was determined by performing the pairwise comparisons between *B. rapa* and *Arabidopsis* G-protein genes. Divergence time was calculated assuming a mutation rate of 1.5 x 10^−8^ substitutions per year [38]. The Ks value for *Gα, Gβ*, and *Gγ* gene homologs were 0.42, 0.43–0.55, and 0.30–0.44, respectively, which indicated that the *B. rapa* G-protein genes, in general, have diverged between 10.08–18.60 mya (Table 2), which correspond well with the whole genome triplication event estimated around 13–17mya in *Brassica* lineage [31]. Interestingly, among the multiple G-protein subunit genes, low Ks value was observed for most of the *BraGγ* gene homologs thereby suggesting that *BraGγ* genes have diverge out significantly and recently (10.08–14.72 mya) during the *Brassica* evolution.

**Table 2:** The synonymous base substitution (Ks) and divergence time estimation of *B. rapa* G-protein genes with corresponding *Arabidopsis* ortholog.

*B. rapa* gene	*Arabidopsis* ortholog	Ks	Ka	Ka/Ks	Divergence time (mya)
*BraA.Gα1*	*AtGPA1*	0.42	0.01	0.04	14.23
*BraA.Gβ1*	*AtAGB1*	0.55	0.03	0.07	18.60
*BraA.Gβ2*	*AtAGB1*	0.46	0.04	0.11	15.46
*BraA.Gβ3*	*AtAGB1*	0.43	0.03	0.07	14.48
*BraA.Gγ1*	*AtAGG1*	0.44	0.10	0.24	14.72
*BraA.Gγ2*	*AtAGG2*	0.33	0.04	0.12	11.14
*BraA.Gγ3*	*AtAGG2*	0.43	0.05	0.12	14.52
*BraA.Gγ4*	*AtAGG3*	0.36	0.10	0.30	12.02
*BraA.Gγ5*	*AtAGG3*	0.30	0.09	0.33	10.08

The divergence time (mya; million years ago) was calculated assuming a mutation rate of 1.5 x 10^−8^ synonymous substitutions per site per year.

Ks- synonymous base substitution; Ka- non-synonymous base substitution.

### Gene structure and genome organization of *B. rapa* G-protein genes

The multiple G-protein subunit sequences identified in *B. rapa* might have originated because of the genome-triplication events common to all *Brassica* diploid species [29], [32], [33]. We therefore analyzed the gene structure, organization of exon and intron, and the genomic location of the nine G-protein genes in *B. rapa* genome, available in BRAD database.

**Figure 3:**
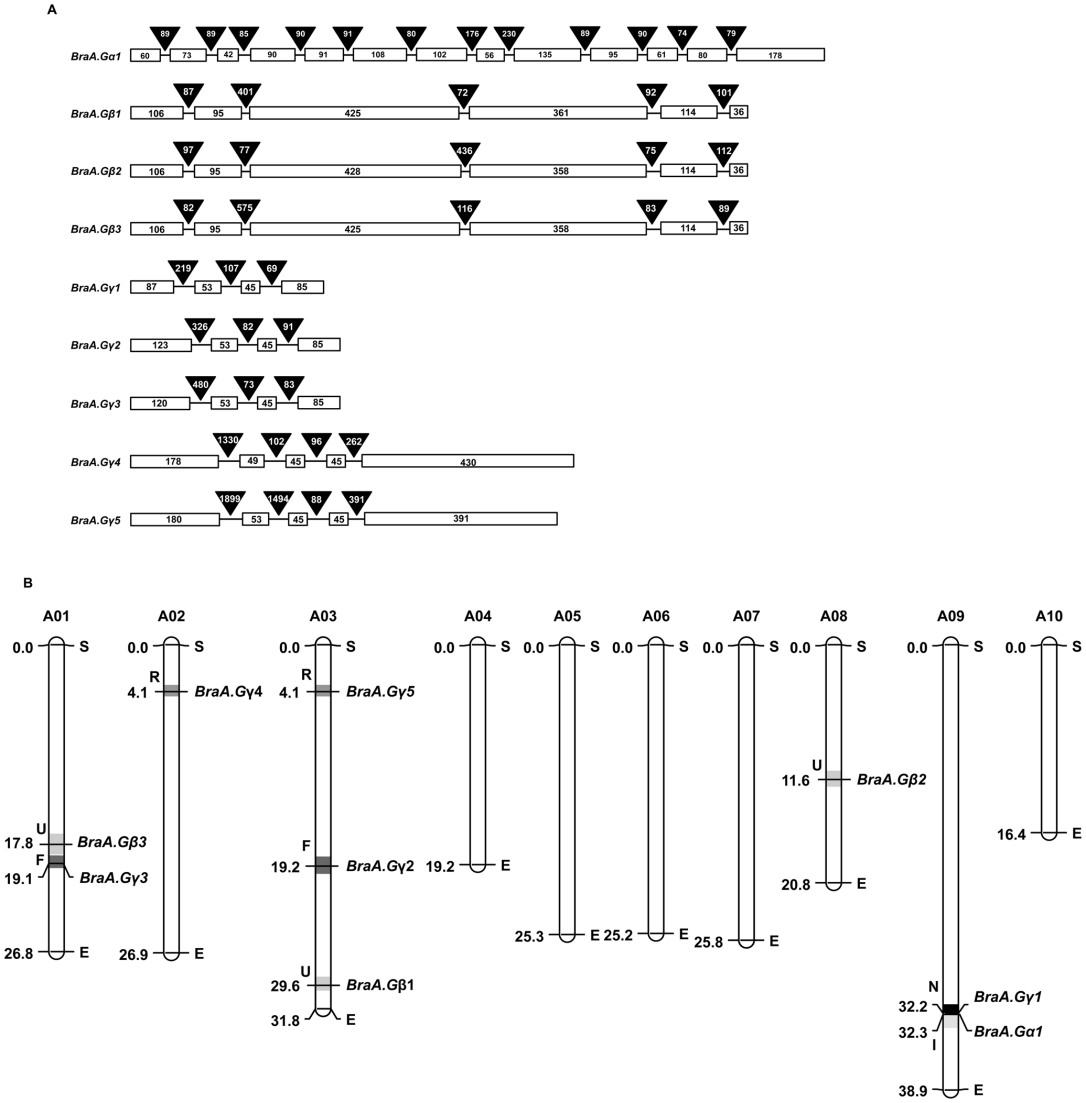
Genomic structure and chromosomal location of *B. rapa* G-protein genes. (**A**) Genomic structure of the identified G-protein subunit genes from *B. rapa* showing arrangement of the exons (open boxes) and introns (line with shaded triangle). Number denotes the length of respective exon and intron. The exons are drawn to scale. (**B**) Map of the *B. rapa* pseudo-chromosomes showing the distribution of the identified G-protein genes of *B. rapa*. The physical location (in Mbp) of G-protein genes is marked on *B. rapa* pseudo-chromosomes based recently available genome browser (chromosome v1.5) available at BRAD database (http://www.brassicadb.org/). S & E denotes the start and end of the chromosomes; and the letters (F, I, N, R and U) with shaded patterns represent the genomic blocks containing the G-protein genes. The pseudo-chromosomes were constructed using MapChart 2.2 (Wageningen).

The canonical 2420 bp full-length *BraA.Gα1* gene (Bra007761) contains 12 introns with the corrected ORF of 1152 bp (Figure 3A). The three full-length *BraGβ* genes (*BraA.Gβ1*, *BraA.Gβ2*, and *BraA.Gβ3*) differ in their length from 1890–2079 bp, with their corresponding ORF size ranging from 1134–1137 bp, which was similar to that amplified in the current study (Figure 3A, Table 1). The *BraGβ* genes contain six exons and five introns. The sizes of exons were conserved, whereas the introns of the *BraGβ* genes were found to differ both in size and sequences from each other, suggesting that the three *BraGβ* genes were originated from a common gene ancestor and have diverged in their intronic sequences during *Brassica* evolution. In contrast, the full-length sequences of the five BraGγ genes (*BraA.Gγ1, BraA.Gγ2, BraA.Gγ3, BraA.Gγ4, and BraA.Gγ5*) were highly divergent in their sizes ranging from 665–4554 bp (Figure 3A, Table 1). The coding sequences of corresponding five *BraGγ* genes amplified in the current study ranged from 270–798 bp. Genomic structure analysis of *BraGγ* genes reveals extreme divergence in their gene structures. The three type-A *Gγ* genes (*BraA.Gγ1, BraA.Gγ2* and *BraA.Gγ3*) contain four exons and three introns; whereas the two the type-C *Gγ* genes (*BraA.Gγ4* and *BraA.Gγ5*) contain five exons and four introns. Interestingly, the length of second and third exons was found to be conserved in all the five *BraGγ* genes, and code for the highly conserved, middle coiled-coil domain of BraGγ proteins, thereby suggesting that the divergent Gγ proteins have originated from this core sequence and acquired variable N- and C-terminal sequences during evolution [3].

We further analyzed the chromosomal location of all nine G-protein genes and mapped them onto the pseudo-chromosomal physical map of *B. rapa* based on recently available information in BRAD database (v1.5). The nine G-protein genes were localized into five out of ten chromosomes, and were interspersed into different sub-genomes of *B. rapa* (Figure 3B, Table 3). The canonical *BraA.Gα1* (Bra007761) was localized in the least fractionized, LF sub-genome (Figure 3B), whereas no *Gα* type sequences was observed in MF1 and MF2 sub-genomes (Table 3). Interestingly, when we analyzed the array of orthologous genes within the common genomic block ‘I’ shared between *A. thaliana* and *B. rapa*, we found that syntenic orthologs of most of the genes occupied within the said genomic block (I) were significantly lost from all three sub-genomes of *B. rapa*, including the LF sub-genome (Table S3). Thus because of gene-fractionation of *B. rapa* sub-genomes only one copy of *Gα* gene was retained in the extant *B. rapa*. The three *Gβ* genes (Bra017658, Bra034628 and Bra011536) were found to be retained in all the three sub-genomes of *B. rapa* within the same genomic block ‘U’ (Figure 3B, Table 3), thereby confirming that the three *BraGβ* subunit genes have evolved from whole genome-triplication events of the single core *Gβ* gene from its Brassicaceae ancestor. Among the *BraGγ* subunit genes, *BraA.Gγ1* (Bra007741), the only ortholog of *AtAGG1* was localized in the LF sub-genome, whereas two orthologs each of *AtAGG2* and *AtAGG3* could be observed in the MF1 and MF2 sub-genomes of *B. rapa* (Table 3). Thus, our analysis clearly suggested that the process of genome-triplication coupled with genome-shrinkage and gene-loss phenomenon [31], have shaped both quantitative and structural divergence of G-protein subunit genes in the extant *B. rapa* genome.

**Table 3:** Summary of syntenic G-protein genes identified in three sub-genomes of *B. rapa* available in BRAD database (http://www.brassicadb.org/).

*Arabidopsis* G-protein genes	Locus ID	Least gene fractionized (LF)	Moderately gene fractionized (MF1)	Most gene fractionized (MF2)
*AtGPA1*	At2g26300	Bra00776 (*BraA.Gα1*)	-	-
*AtAGB1*	At4g34460	Bra011536 (*BraA.Gβ3*)	Bra017658 (*BraA.Gβ1*)	Bra034628 (*BraA.Gβ2*)
*AtAGG1*	At3g63420	Bra007741 (*BraA.Gγ1*)	-	-
*AtAGG2*	At3g22942	-	Bra023782 (*BraA.Gγ3*)	Bra001894 (*BraA.Gγ2*)
*AtAGG3*	At5g20635	-	Bra006568 (*BraA.Gγ5*)	Bra020117 (*BraA.Gγ4*)

### Tissue specific expression of *B. rapa* G-protein genes across developmental stages

**Figure 4:**
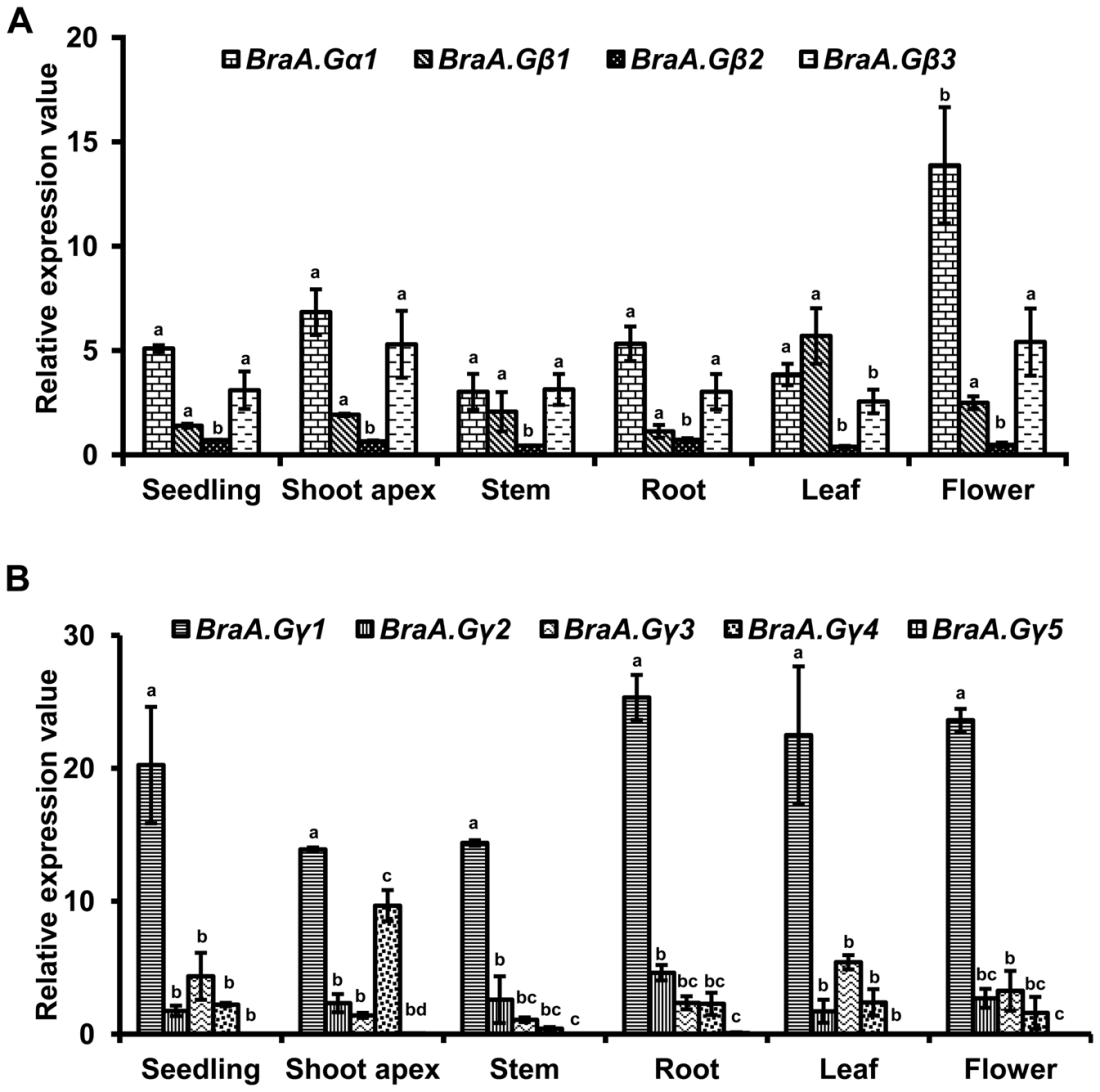
Expression pattern of G-protein genes in different tissues of *B. rapa*. Relative transcript accumulation of (**A**) *BraA.Gα1* and *BraGβ* genes; and (**B**) *BraGγ* genes across different tissue types of *B. rapa*. Total RNA was extracted from seedling, shoot apex, stem, root, leaf, and flower; and relative expression of G-protein genes was quantified using qRT-PCR analysis and normalized against *Brassica Actin* gene expression (set at 100). Each experiment was performed with three biological replicates and the data were averaged. Bar represent the standard error of the mean. Different letters above the bar represent the significant difference at P<0.05 using Fisher's LSD test, among the homologs of each G-protein subunit genes.

The multiplicity of G-protein genes in *B. rapa* led us to perform their detailed expression analysis, using qRT-PCR analysis, in various plant tissue types representing different developmental stages of *B. rapa*. All the nine G-protein subunit genes were ubiquitously expressed across various developmental stages of *B. rapa* (Figure 4), as also reported in other plant species [4], [5], [42]–[44]. In general, the canonical *BraA.Gα1* and the three *BraGβ* genes showed comparable transcript abundance across different tissue types of *B. rapa*. The *BraA.Gα1* was expressed throughout the plant development, with highest expression observed in flower and shoot apex (Figure 4A). Among three *BraGβ* genes, *BraA.Gβ1* and *BraA.Gβ3* genes showed comparably higher transcript abundance, whereas *BraA.Gβ2* was the least expressed transcript across all the tissue types tested (Figure 4A). A similar trend was also observed for the *BraGγ* genes, wherein the expression of *BraA.Gγ1* gene was found to be the highest, although other *BraGγ* genes showed lower and comparable expression pattern (Figure 4B). Overall, the G-protein genes showed ubiquitous but distinct expression pattern across different tissue types representing developmental stages of *B. rapa*.

**Figure 5:**
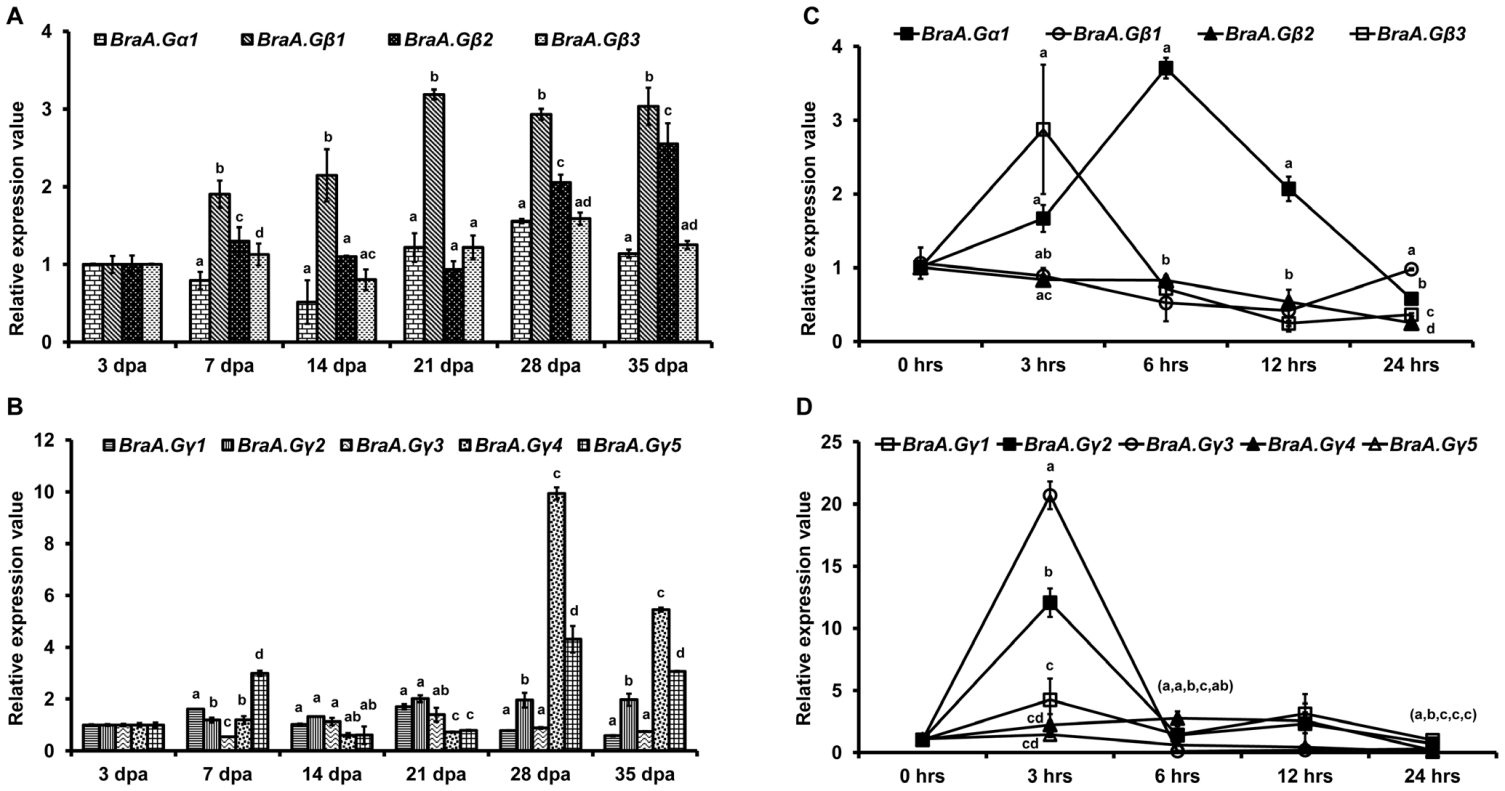
Expression pattern of *B. rapa* G-proteins during seed maturation (A, B) and seed germination (C, D). The relative expression of (**A, C**) *BraA.Gα1* and *BraGβ* genes; and (**B, D**) *BraGγ* genes across different stages of seed-maturation (3, 7, 14, 21, 28, and 35 days post-anthesis; dpa), and seed-germination (0, 3, 6, 12 and 24 hrs post-imbibitions) of *B. rapa*. Total RNA was isolated from different tissues and the relative expression was quantified by qRT-PCR analysis and normalized against *Brassica GAPDH* (for seed-maturation) and *Actin* (for seed-germination). The (**A, B**) 3 dpa immature seeds and (**C, D**) 0 hrs (un-imbibed) seeds were used as reference tissue (expression set at 1). Three independent measurements were taken and the data were averaged. Bar represent the standard error of the mean. Different letters above represent significant difference at P<0.05 using Fisher's LSD test. Letters within the parenthesis represent significance for *BraA.Gγ1-BraA.Gγ5*, in series.

Genetic studies in model plant *Arabidopsis* and in crops such as rice and soybean indicated that G-protein play important roles during seed maturation and germination [4], [45]. Seeds are the most economically important component of *B. rapa* plants, as they are associated with both food and feed values. We therefore investigated the detailed expression profile of individual G-protein genes during different stages of seed maturation and germination. Real time qRT-PCR expression analysis showed that all the nine G-protein genes were expressed throughout the seed maturation stages (Figure 5). The canonical *BraA.Gα1* genes retained almost constant expression pattern, whereas the overall transcript accumulation of *BraGβ* and *BraGγ* genes was found to be comparably higher during seed maturation (Figure 5). As also observed across plant developmental stages (Figure 4), the multiple homologs of *BraGβ* and *BraGγ* genes showed distinct expression profile across seed maturation stages. For example, *BraA.Gβ1* genes showed comparably higher transcript accumulation throughout the seed development stages, whereas the expression of *BraA.Gβ2* increased during the later phases of seed maturation thereby indicating differential expression pattern among *BraGβ* genes (Figure 5A). Similarly, among *BraGγ* genes, the expression of both type-C *Gγ* genes (*BraA.Gγ4* and *BraA.Gγ5*) was specifically up-regulated during later stages of seed maturation (Figure 5B), suggesting their key involvement with seed related traits [23], [24].

We also investigated the expression profiles of G-protein genes during seed germination stages in *B. rapa*. Most of the G-protein subunit genes showed increased accumulation of transcripts during early time points of seed germination, albeit at different level (Figure 5C). The accumulation of canonical *BraA.Gα1* transcript was found to be increased significantly within 6 hrs of seed imbibitions (Figure 5C). Although such up-regulation was also observed for *BraGβ* and *BraGγ* genes, different homologs of these genes showed differential up-regulation expression pattern. For example, among three *BraGβ* genes, only *BraA.Gβ3* showed up-regulation of transcripts during very early time point of seed germination. Differential up-regulation was also observed for *BraGγ* genes (Figure 5D), wherein *BraA.Gγ3, BraA.Gγ2* and *BraA.Gγ1* genes, in order, showed significant increase in their transcript accumulation during early stages of seed-germination, whereas transcripts of type-C *Gγ* genes (*BraA.Gγ4* and *BraA.Gγ5*) remained comparably lower throughout the germinating seed stages (Figure 5D). All the G-protein genes retained almost similar expression profile around 24 hrs of seed-imbibitions. The differential transcript accumulation of G-protein subunit genes in all possibility suggests their expression differentiation and distinct roles towards plant growth and development processes, including seed maturation and germination, in mesohexaploid *B. rapa*.

### Transcriptional regulation of G-protein genes in response to phytohormone and stress treatments

**Figure 6:**
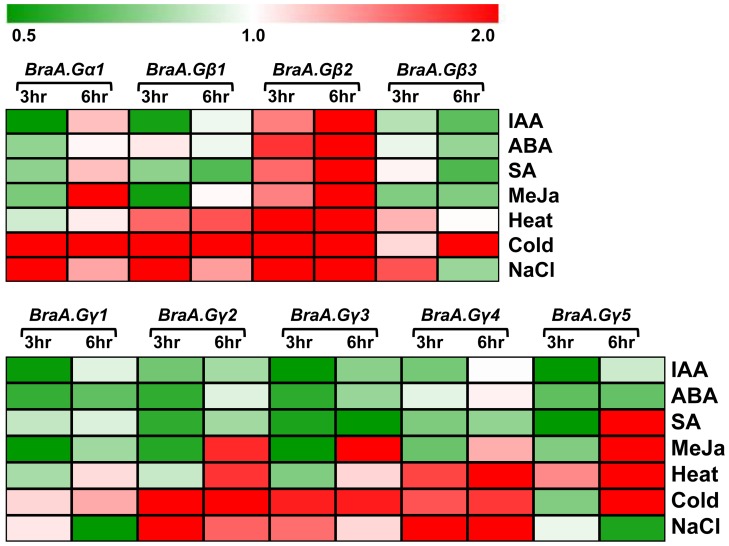
Heat map showing transcript accumulation of G-protein genes in response to phytohormone and stress conditions. Relative expression of (**A**) *BraA.Gα1* and *BraGβ* genes; and (**B**) *BraGγ* genes in five days seedlings treated with various phytohormones and stress elicitors. The relative expression of G-protein genes was quantified by qRT-PCR analysis and normalized against *Brassica Actin* (set at 1). The averaged data of three independent biological replicates was used to plot the heat map.

G-proteins are known to interact with various hormone signaling pathways and also play key roles under various stress conditions in plants [9], [15]–[17]. To examine the transcriptional regulation of *B. rapa* G-protein genes, we analyzed the expression of these genes on five day old seedlings treated with various plant elicitors including phytohormones and conditions mimicing abiotic and biotic stresses, on time dependent manner. The *B. rapa* G-protein genes were altered differentially in response to various stress conditions (Table S4). The *BraA.Gα1* showed a higher induction (>2 fold) of transcripts in response to cold, salt and MeJa treatments, compared to that observed for SA, IAA, ABA and heat treatments (Figure 6). Intrestingly, *BraA.Gβ2* which was otherwise the least abundant transcript among *BraGβs* across developmental tissues, showed an early and significant (ranging from 4.32 to 13.91 fold) induction of transcript in response to all the biotic and abiotic conditions and phytohormone treatments (Figure 6; Table S4), thereby suggesting key involvement of the *BraA.Gβ2* under various stress conditions. The *BraA.Gβ1* and *BraA.Gβ3* (to some extent) were only up-regulated in response to abiotic stress conditions including heat, cold and salt treatments.

The *BraGγ* genes also showed differential induction of transcripts in response to most of the stress and phytohormone treatments (Figure 6). In general, the expression of all *BraGγ* genes was mostly induced in response to abiotic stress conditions including altered temeratures and salt treatment, although at different levels. In response to abiotic stress conditions, the expression of *BraA.Gγ2* and *BraA.Gγ4* was induced significantly during early time points, whereas expression of *BraA.Gγ1*, the highly abundant transcript across various develpmental tissues (Figure 4B), was least affacted. In reponse to SA and MeJa treatments, which mimic the biotic stress conditions, the expresion of *BraA.Gγ5* gene was highly upregulated, whereas *BraA.Gγ2*, *BraA.Gγ3* and *BraA.Gγ4* (to some extent) showed induction of their transcripts only in response to MeJa treatment. The expression of *BraGγ* genes was not found to be altered in response to the exogenously treated phytohormones including ABA and IAA. Such diffrential transcriptional regulation of multiple G-protein subunit genes in response to various phytohomrome and stress treatments in all possibility suggest the involvement of condition-specific heterotrimeric combination(s) in mesohexaploid *B. rapa*.

### Protein-protein interactions between G-protein subunits of *B. rapa*

**Figure 7:**
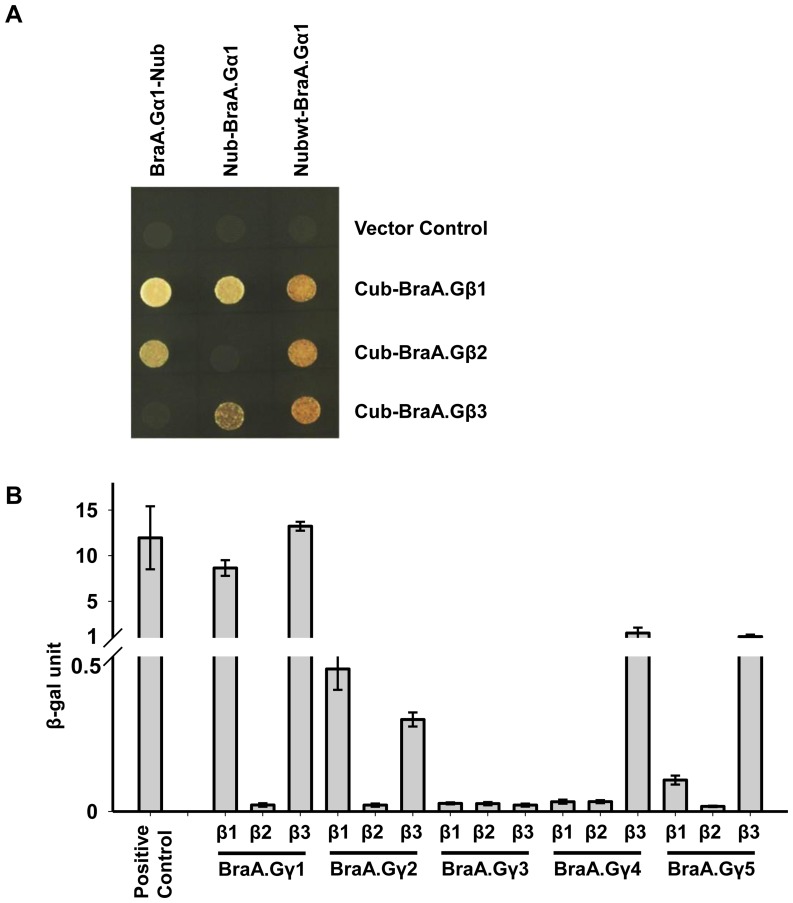
Interaction between *B. rapa* G-protein subunits. (**A**) Interaction between BraA.Gα1 and BraGβ proteins using split ubiquitin-based interaction assay. The picture shows yeast growth on selective media containing 500 µM methionine. In all cases, Gα protein was used as Nub (N-terminal half of ubiquitin) fusion in both orientations, and Gβ proteins were used as Cub (C-terminal half of ubiquitin) fusions. Nub_wt_ (N-terminal half of wild-type ubiquitin)-Gα1 fusion construct was used as positive control for interaction. Two biological replicates of the experiment were performed with identical results. (**B**) Interaction between BraGβ (in pDEST32) and BraGγ (in pDEST 22) proteins using yeast-two-hybrid assay. The colorimetric assay based on ONPG substrate was performed thrice, independently, and the data were averaged. Error bars represent the standard error of the mean. Positive control refers to the interaction strength of pDEST32-RalGDS-wt with pDEST22-Krev1 vector.

To check the strength and specificity of interactions between multiple heterotrimer combinations of *B. rapa* G-protein subunits, we performed protein-protein interaction screens in yeast. Interaction of the canonical BraA.Gα1 protein with BraGβ subunits, in all possible combinations, was assayed using mating based split ubiquitin system (mbSUS), wherein BraA.Gα1 was used as prey, and BraGβs were used as bait proteins. All three BraGβ proteins showed interaction with BraA.Gα1 protein (Figure 7A), although at variable strength and specificity. Among all the three BraGβs, BraA.Gβ1 showed very high level of interaction with BraA.Gα1, in both orientations, even at high methionine concentration. However, BraA.Gβ2 and BraA.Gβ3 proteins, independently, could interact with BraA.Gα1 in only one of the two possible orientations, thereby suggesting their relatively lower interaction specificity with BraA.Gα1 protein.

To check the interaction between BraGβ and BraGγ proteins, yeast two hybrid assay was performed. BraGβ and BraGγ, independently, were used as bait and prey proteins, respectively, and β-galactosidase activity assay was used to determine the quantitative difference of interactions. The BraGβ and BraGγ subunits showed visible interaction specificities when tested in yeast (Figure 7B). In general, both BraA.Gβ1 and BraA.Gβ3 showed a higher level of interaction with BraGγ proteins, compare to BraA.Gβ2 showing weak interaction with all BraGγ proteins. Further, among BraGγ proteins, BraA.Gγ1 was found to have the strongest interaction with BraGβs. Both BraA.Gγ4 and BraA.Gγ5 proteins showed a high level of interaction with BraA.Gβ3 protein only. The interaction specificity among multiple BraGβ and BraGγ proteins was also confirmed using the growth based assay utilizing higher concentrations of 3AT, a competitive inhibitor of HIS3 (Figure S5). Thus protein-protein interaction studies clearly showed a high level of interaction specificity among multiple G-protein subunits in mesopolyploid *B. rapa*.

## Discussion

Heterotrimeric G-protein is one of the key signal transduction components which are known to regulate a wide range of biological functions across plants and metazoans. Previous reports have shown that there exists a huge quantitative disparity between G-protein components between plants to animals [2], [45]. In contrast to animal system, most of the species belonging to land plants have limited repertoire of conventional G-protein subunit genes. However, with extensive polyploidy across plant kingdom, the multiplicity of G-protein components is quite expected, which indicates that plant kingdom also uses more diverse and complex G-protein signaling network to control various biological processes.

### Evolution and sequence divergence of G-protein subunit genes in *B. rapa*

The genome of model plant *Arabidopsis* has one canonical Gα (AtGPA1) and Gβ (AtAGB1) subunits and three Gγ (AtAGG1, AtAGG2, and AtAGG3) subunits encoding genes [3], [7]. While investigating the inventory of G-protein subunit genes in one of its taxonomically closest crop, *B. rapa*, we have identified a single homolog of plant *Gα* (*BraA.Gα1*), three homologs of *Gβ* (*BraA.Gβ1, BraA.Gβ2* and *BraA.Gβ3*) and five homologs of *Gγ* (*BraA.Gγ1, BraA.Gγ2, BraA.Gγ3, BraA.Gγ4* and *BraA.Gγ5*), which constitute the most elaborate G-protein signaling network from any *Brassica* crop, known to date (Table 1). Besides, it is the second largest G-protein family known from any land plant next to soybean genome which encodes four Gα, four Gβ and ten Gγ proteins [4], [5].

*B. rapa* is a mesohexaploid (or paleohexaploid) species with complex genome architecture and there are evidences that *B. rapa* have evolved from whole genome triplications events, after its divergence from *A. thaliana* from a common diploid Brassicaceae ancestor [29]. As a result, *B. rapa* genome harbours three sub-genomes namely least gene fractionized (LF), moderately gene fractionized (MF1) and most gene fractionized (MF2) subgenomes, thereby creating a possibility of up to three copies of each of the gene from its ancestral genome. Our data suggest that the G-protein subunit genes in *B. rapa* are shaped differentially during evolution. Although, whole genome triplication events have proportionally increased the catalog of *Gβ* genes (three homologs) in mesohaxaploid *B. rapa*, but only one *Gα* gene (*BraA.Gα1*), and 1–2 homologs of each of the three *Arabidopsis Gγ* genes could be amplified from *B. rapa* genome. Even across morphologically and genetically divergent cultivars of *B. rapa*, only one *Gα* gene sequence was identified in the current study. Interestingly, as also observed in *B. rapa*, a single member of canonical *Gα* gene is present in related *Brassica* species including *B. oleracea* (Bol044641) and *B. napus* [46], sharing significantly high level of sequence identity. Comprehensive scan of G-protein gene sequences into *B. rapa* genome assembly further confirms that the homologs of *Gβ* gene are uniformly present in all the three, namely LF, MF1 and MF2 sub-genomes of *B. rapa*, whereas homologs of *Gα* and *Gγ* genes are present only in one and two sub-genomes of *B. rapa*, respectively (Table 3; Figure 3). This in all possibility suggest that few homologs of *BraGα* and *BraGγ* genes might have lost due to extensive gene-loss or gene fractionation phenomenon that occurred during the evolution of *B. rapa* [31].

Amino acid sequence analysis of canonical Gα protein present across members of Brassicaceae family, including *B. rapa, B. oleracea*, *B. napus* and *Arabidopsi*s, suggest that Gα protein have retained extensive domain conservation and its core structure (Figure 1A), thereby suggesting that Gα proteins might have maintained functional conservation, at least within Brassicaceae family. Similarly, the three BraGβ proteins, originated via whole genome triplication events of a common gene ancestor, have retained all the necessary domains and residues required for interaction with Gα and Gγ proteins (Figure 1B). However, over time, the sequence of the duplicated *BraGβ* and *BraGγ* genes have diverge from each other, as a consequence of which the duplicated genes might have undergone functional divergence, including neo-functionalization and sub-functionalization [47], [48].

Gγ proteins are an integral part of G-protein heterotrimer, required for proper targeting of Gβ subunit and of intact heterotrimer to the plasma membrane. Sequence analysis across plant kingdom suggests that Gγ proteins are the most abundant and diverse of the three G-protein subunits having a low level of sequence and structural identity among themselves. In general, the plant Gγ proteins are sub-divided into three types based on their C-terminus region namely, type-A Gγ protein with C-terminus CaaX motif; type-C Gγ protein containing highly enriched cysteine residues at their C-terminal end; whereas the type-B Gγ protein lacks both CaaX and the cysteine residues at C-terminus end [3], [5]. Based on C-terminal region, we could identify three type-A Gγ (BraA.Gγ1, BraA.Gγ2 and BraA.Gγ3) and two type-C Gγ (BraA.Gγ4 and BraA.Gγ5) proteins in *B. rapa*. Amino acid sequence alignment revealed that the central region of both types of BraGγ proteins was most conserved, while these proteins have acquired variable N- and C-terminal sequences during evolution. We presume that the presence of divergent BraGγ proteins, along with multiple BraGβ proteins, in all possibility controls the functional selectivity of the G-protein heterotrimer in *B. rapa*, as also reported for the three diverse Gγ proteins of *Arabidopsis* [3], [43].

G-protein coupled receptor (GPCR), a seven trans-membrane integral protein, is one of the apex components in G-protein signaling, which perceive external signals and transduces it to the cell interior by interacting with Gα subunit of the G-protein heterotrimer [1], [2]. Although more than 800 GPCRs have been reported in human for performing multiple diverse cellular processes, only four GPCRs are identified in model plant Arabidopsis, namely GCR1 (At1g48270), GCR2 (At1g52920), GTG1 (At1g64990) and GTG2 (At4g27630). When we investigated the repertoire of GPCRs in polyploid *B. rapa* genome (http://www.brassicadb.org/) only one homolog each of GCR1 (*B. rapa* ID: Bra018719), GCR2 (Bra019001), GTG1 (Bra022501) and GTG2 (Bra026330) was identified. The *Brassica* homologs of all GPCRs, except for disputed GCR2, share high level (>90%) of amino acid sequence identity with their *Arabidopsis* counterpart. It is interesting to note that although, whole genome triplication event has resulted in the multiplicity of most of the genes in *Brassica* lineage, the inventory of interacting BraGPCRs and BraGα is selectively reduced and remained conserved in the extant *B. rapa* genome, thereby suggesting that the divergent *BraGβ* and *BraGγ* subunits are the key components controlling G-protein signaling diversity in mesopolyploid *B. rapa*.

### Expression divergence and developmental regulation of *B. rapa* G-protein genes

Studies in polyploids have shown that duplicated (homoeologeous) genes may have alterations in the expression, such as differential expression pattern, transcriptional bias or gene silencing of homoeologs in various organs of the plant or in response to various environmental stimuli [48]–[50]. The majority of these alterations are known to be caused by *cis*-regulatory divergence between the homoeologous genes, thereby giving rise to transcriptional sub-functionalization.

The presence of multiple copies of each G-protein genes in *B. rapa* provides an opportunity to assess their specific expression patterns as a consequence of whole genome-triplication events in *B. rapa*. We found that multiple G-protein subunit genes of *B. rapa* have ubiquitous, overlapping but distinct expression profiles across plant development and in response to various stress conditions. For example, both *BraA.Gβ1* and *BraA.Gβ3* have higher transcript abundance among *BraGβ* genes across various developmental tissue types, whereas *BraA.Gβ2* transcripts are highly upregulated during both biotic and abiotic stress condition. It is interesting to note that *BraA.Gβ2* is phylogeneticaly closer to the reported *Gβ* subunit gene of *B. napus*, which was previously shown to be responsive to abiotic stress [51], thereby suggesting expression conservation of *Gβ* genes across *Brassica* species. Similarly, *BraA.Gγ1* is the highly abundant transcript among *BraA.Gγ* genes across all the tissue type tested, whereas other *BraGγ* genes were preferentially up-regulated during abiotic and biotic stress conditions, indicating their possible roles in stress signaling including providing resistance against plant pathogens, as also reported earlier for *Arabidopsis AGG1* [20].

In recent years, the type-C Gγ genes have been shown to regulate organ development, seed size and shape and oil production in plants [23], [24], [26]. In current study, both type-C *Gγ* genes (*BraA.Gγ4* and *BraA.Gγ5*) along with two *BraGβ* genes (*BraA.Gβ1* and *BraA.Gβ2*) were found to be up-regulated during later stages of seed-maturation, thereby suggesting that these genes might have key role in seed development processes in oilseed *B. rapa*. Similarly, during the early stages of seed germination, the expression level of G-protein genes including *BraA.Gα1*, *BraA.Gβ3*, and all three type-A *BraGγ* genes increases significantly, thereby suggesting that G-protein gene play a key role during seed germination process, possibly by interacting with plant hormone signaling pathways, as also reported earlier in *Arabidopsis* and soybean [4], [9]. Such high degree of expression partitioning of multiple G-protein genes across different tissues and stress treatments in all possibility suggests the involvement of tissue- and condition-specific heterotrimer and heterodimer in controlling various developmental processes in mesphexaploid crop *B. rapa* [4], [5].

To confirm the differential transcriptional regulation of G-protein genes, we analysed the 5′ regions (1.5 kb upstream to ATG) of each G-protein gene. The 5′ upstream sequences of *B. rapa* G-protein genes was found to be highly divergent among themselves (Table S5, Figure S2-S4). Further, when we queried the 5′ upstream region in the PLACE database, all the nine G-protein genes of *B. rapa* contained tissue specific *cis*-regulatory elements like (CAATbox1), transcriptional activator like ARR1AT, regulatory element involved in plant defense like WRKY71OS, transcription factors like DOFCOREZM, and MYC recognition site (MYCONSENSUSAT) (Table S6). Various stress related *cis*-elements such as ABA responsive elements-ABRE, dehydration responsive elements, were also present in the 5′ upstream region of G-protein genes. We presume that the quantitative disparity of various *cis*-regulatory elements present in the 5′ upstream region of *B. rapa* G-protein genes possibly explains their differential expression pattern across plant development and various environmental conditions.

### *B. rapa* G-protein subunits have interaction specificity

In metazoans, the multiplicity of G-protein subunits and G-protein coupled receptors (GPCRs) provides a complex signal transduction architecture, wherein specific GPCR-Gαβγ combinations are known to transduce specific extracellular signals within cells [1]. The presence of multiple G-protein subunits in *B. rapa* led us to investigate the specificity of subunit interaction using yeast-based interaction assays. The *B. rapa* G-protein subunits interacted with each other in most of the possible combinations however, a high degree of interaction specificity between the multiple G-protein subunits was also observed. Although all the three BraGβ proteins shared a very high level of amino-acid sequence identity and contained all the necessary residues required for the interaction with Gα [41], BraA.Gβ1 protein interacted strongly with the canonical BraA.Gα1 (Figure 7A). It is quite possible that the divergent residues acquired among BraGβ proteins during the evolution could direct the interaction specificity, and the development of tissue- or condition-specific G-protein heterotrimer.

The BraGβ and BraGγ proteins also interacted with each other showing strong interaction specificity, which could also be attributed to the presence of vast sequence diversity across BraGγ proteins (Figure 7B), and to the less divergent BraGβ proteins to some extent. Among the Gγ proteins, BraA.Gγ1 and BraA.Gγ2 showed strong interaction with both BraA.Gβ1 and BraA.Gβ3 but weak interaction with BraA.Gβ2. Both type-C Gγ proteins (BraA.Gγ4 and BraA.Gγ5) showed strong and exclusive interaction with BraA.Gβ3 protein. We presume that under optimal growth conditions and across developmental tissues where certain G-protein transcripts such as *BraA.Gβ1, BraA.Gβ3*, *BraA.Gγ1*, *BraA.Gγ4* and *BraA.Gγ5* are abundantly expressed, these might form the predominant functional G-protein heterotrimer, as also suggested by their strong interaction selectively. However, under stress conditions, a higher expression of other G-protein transcripts such as *BraA.Gβ2*, *BraA.Gγ2*, *BraA.Gγ3*, *BraA.Gγ4* and *BraA.Gγ5* (to some extent) suggest that these might serve as accessory yet important functional G-protein heterotrimer combinations in *B. rapa*. Thus, in polyploid crops where multiplicity and redundancy of G-protein subunit proteins exist, the subunit-specific interaction might be a key way to control the functional selectivity of G-protein heterotrimer in different cell and tissue-types or in response to different stress conditions. The identification of an elaborate and diverse G-protein gene family from *B. rapa* is a first step towards accessing the core G-protein components from economically important *Brassica* crops. Molecular and functional characterization of more G-protein sequences from related *Brassica* species will help us to understand the evolution of G-protein signaling in Brassicaceae family, and vis-à-vis elucidate the role of these key signaling components toward controlling various developmental processes and the agronomically important traits in *Brassica* crops.

## Supporting Information

Figure S1 **Nucleotide sequence alignment of the reported (Bra007761) and amplified** ***BraA.Gα1*** **coding DNA sequences along with their translated amino-acid sequences.**
(PDF)

Figure S2 **Nucleotide sequence alignment of 5′ upstream regions of** ***BraA.Gα1*** **(Bra007761) with** ***AtGPA1*** **.**
(PDF)

Figure S3 **Nucleotide sequence alignment of 5′ upstream regions of** ***BraGβ*** **genes.**
(PDF)

Figure S4 **Nucleotide sequence alignment of 5′ upstream regions of** ***BraGγ*** **genes.**
(PDF)

Figure S5 **Interactions between BraGβ and BraGγ proteins determined by performing yeast-two-hybrid based growth assay.**
(PDF)

Table S1 **List of primers used in the current study.**
(PDF)

Table S2 **Amino acid sequence identity (%) of** ***B. rapa*** **Gα, Gβ and Gγ proteins with corresponding proteins from** ***Arabidopsis thaliana*** **(** ***At*** **) and rice (** ***Os*** **).**
(PDF)

Table S3 **Syntenic flanking genes of** ***BraA.Gα1*** **(Bra007761) present in the chromosomal block ‘I’ of** ***B. rapa*** **, as available in BRAD database (** http://www.brassicadb.org/ **).**
(PDF)

Table S4 **Relative fold expression of the G-protein genes under various phytohormones and stress treatments at different time points.**
(PDF)

Table S5 **Nucleotide sequence identity (in %) of 5′ upstream region (1.5 kb of ATG) of** ***B. rapa*** **G-protein genes with corresponding** ***Arabidopsis*** **G-protein promoters.**
(PDF)

Table S6 **Summary of** ***cis*** **-regulatory elements present in the 5′ upstream region of the G-protein genes of** ***B. rapa*** **.**
(PDF)
