# Supplementary material for: Evolution, Expression Differentiation and Interaction Specificity of Heterotrimeric G-Protein Subunit Gene Family in the Mesohexaploid Brassica rapa
Source: PLoS One. 2014 Sep 5;9(9):e105771. doi: 10.1371/journal.pone.0105771 (PMC4156303; doi:10.1371/journal.pone.0105771)
Supplement: Figure S3 — Nucleotide sequence alignment of 5′ upstream regions of BraGβ genes. (PDF) [file pone.0105771.s003.pdf]

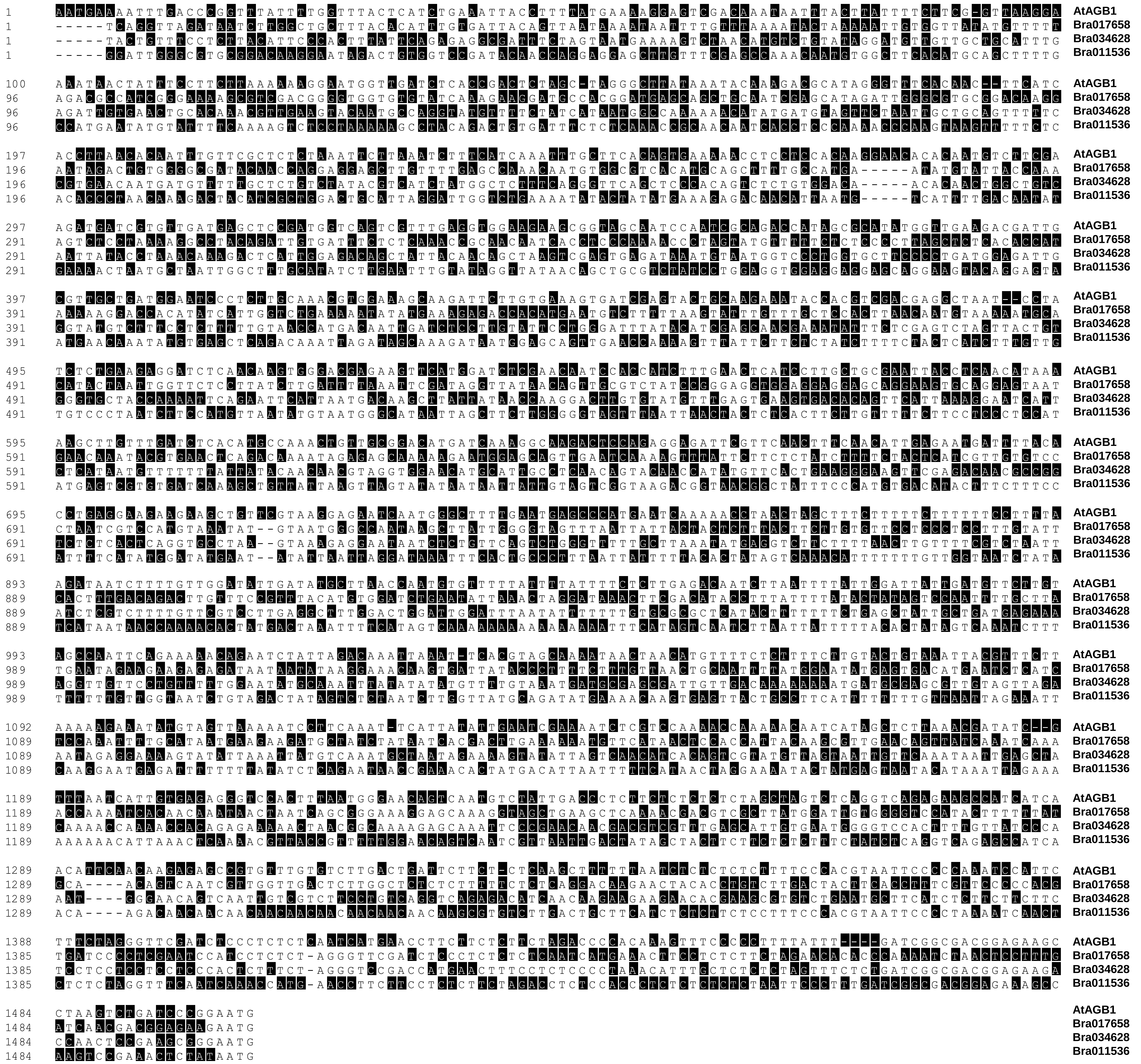

Supplementary Figure S3. Nucleotide sequence alignment of 5' upstream regions of *BraGβ* genes. Sequence alignment of 1.5 kb sequence upstream to putative translation start site (ATG) of *BraA.Gβ1* (Bra017658), *BraA.Gβ2* (Bra034628), *BraA.Gβ3* (Bra011536) along with Arabidopsis *AtAGB1* was performed using Clustal W. The residues differing from consensus are marked in dark background.
