# Supplementary material for: Evolution, Expression Differentiation and Interaction Specificity of Heterotrimeric G-Protein Subunit Gene Family in the Mesohexaploid Brassica rapa
Source: PLoS One. 2014 Sep 5;9(9):e105771. doi: 10.1371/journal.pone.0105771 (PMC4156303; doi:10.1371/journal.pone.0105771)
Supplement: Figure S5 — Interactions between BraGβ and BraGγ proteins determined by performing yeast-two-hybrid based growth assay. (PDF) [file pone.0105771.s005.pdf]

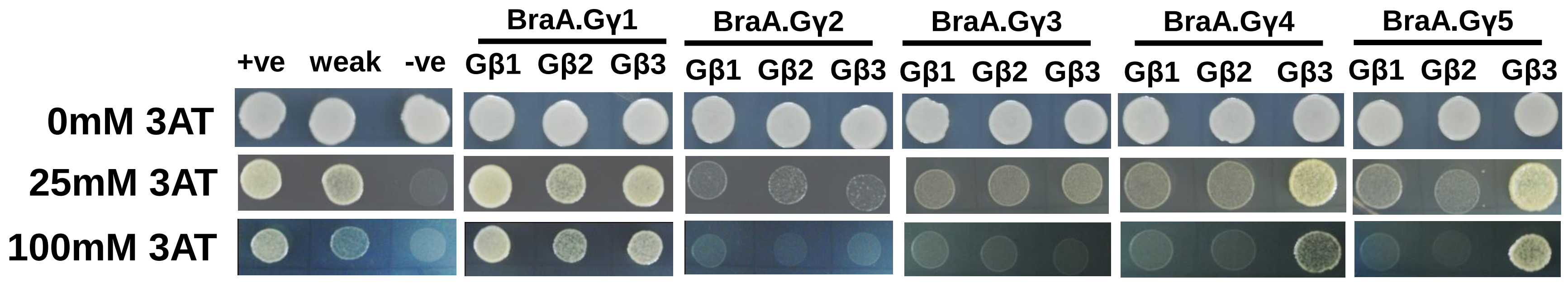

Supplementary Figure S5: Interactions between BraGβ and BraGγ proteins determined by performing yeast-two-hybrid based growth assay. Growth of the diploid yeast cells on minimal media lacking His, Leu and Trp but containing different concentrations of 3AT (25 mM and 100 mM). Positive (+ve), weak and negative (-ve) refers to the interaction of pDEST32-RalGDS-wt with pDEST22-Krev1, pDEST32-RalGDS-m1with pDEST22-Krev1 and pDEST32-RalGDS-m2 with pDEST22-Krev1, respectively.
