# Supplementary material for: Evolution, Expression Differentiation and Interaction Specificity of Heterotrimeric G-Protein Subunit Gene Family in the Mesohexaploid Brassica rapa
Source: PLoS One. 2014 Sep 5;9(9):e105771. doi: 10.1371/journal.pone.0105771 (PMC4156303; doi:10.1371/journal.pone.0105771)
Supplement: Table S1 — List of primers used in the current study. (PDF) [file pone.0105771.s006.pdf]

Supplementary Table S1. List of primers used in the current study.

	Name of Primer	Sequence (5'- 3')
**Primers used for gene amplification**		
	BrGα1 DTopo FP	CACCATGGGCTTACTCTGCAGTAG
	BrGα1 D Topo RP(S)	TCATAAAAGGCCAGCCTCCA
	BrGβ (1-3) DTopo FP	CACCATGTCTGTCTCCGAGCTC
	BrGβ (1-3) DTopo RP(S)	TCATATCACTCTCCTGTGCCCTCCA
	BrGγ1 D Topo FP	CACCATGGAATTGGAAGATGGTGATTCACGA
	BrGγ1 D Topo RP (S)	TCAAAGTATAAAGCATCTGCAGCS
	BrGγ2 D Topo FP	CACCATGATGGAAGCGGGTAGCTCCAATTCG
	BrGγ2 D Topo RP (S)	TTAAAGAATGAAGCAGCCGCATCCT
	BrGγ3 D Topo FP	CACCATGGAAGTGGGTAGCTSSAATTCGTC
	BrGγ3 D Topo RP (S)	TTAGTTAGAGAATGAAGCATCCACATCGT
	BrGγ4 D Topo FP	CACCATGGCTTCTACTTCATGCAGTGTCAA
	BrGγ5 D Topo FP	CACCATGTCTGCTCCTTCATGTAGTGTCGG
	BrGγ(4-5) D Topo RP(S)	TTAGAAAGCTAAACAACAAGGAT
**Primers used for Quantitative Real time PCR**		
	BrGα1 FP (RT)	GAGAATTTGAAGAGATTATCAGATGTC
	BrGα1 RP (RT)	AATAACAGCAGTAACACCTTCAAAC
	BrGβ1 FP (RT)	ATCAGCTCCAAGTGTATCATCAG
	BrGβ1 RP (RT)	TATCCCAAACGTAGCAAGTGC
	BrGβ2 FP (RT)	TGGTCATCAACTCCAGGTGTAC
	BrGβ2 RP (RT)	TCTTATGTGAATCCTGAAGCTG
	BrGβ3 FP (RT)	TTCATTTCTGGTTCATGCGAC
	BrGβ3 RP (RT)	CGTTCTCACCATCACCATGTG
	BrGγ1 FP (RT)	TTCTTGGAGAAAGAGTTGGAAG
	BrGγ1 RP (RT)	TCAAAGGCCCATTGGTCAGT
	BrGγ2 FP (RT)	ATGCCGAACTCAAAAGGCTT
	BrGγ2 RP (RT)	AACCATTGATCCCATGTTCCA
	BrGγ3 FP (RT)	GCTTCTTAGAGGAAGAGCTAGAGCT
	BrGγ3 RP (RT)	GAGAATGAAGCATCCACATCG
	BrGγ4 FP (RT)	TGTCGAAGGCTTGCAACCGTCG
	BrGγ4 RP (RT)	GGCAACAACAGCAGAAACTCGCCAC
	BrGγ5 FP (RT)	CATCCAAGGGGTACAACCGGCT
	BrGγ5 RP (RT)	GCTCAAACACGGAATACCG
	BrACT FP (RT)	TGACAATGGAACTGGATGGT
	BrACT RP(RT)	TAGACTGAGCTTCATCACCAAC
	BrGAPDH FP (RT)	TCAGTTGTTGACCT CACGGTT
	BrGAPDH RP (RT)	CTGTCACCAACGAAGTCAGT
**Primers used for amplification of Split Ubiquitin interaction**		
	BrGα1 SUS FP	ACAAGTTTGTACAAAAAAGCAGGCTCTCAACCACCATGGGCTTACTCTGCAGT
	BrGα1 SUS RP	TCCGCCACCACCAACCACTTTGTACAAGAAAGCTGGGTATAAAAGGCCAGCYTCCAA
	BrGβ(1-3) SUS FP	ACAAGTTTGTACAAAAAAGCAGGCTCTCCAACCACCATGTCTGTCTCCGAGCTCA
	BrGβ(1-3) SUS RP	TCCGCCACCACCAACCACTTTGTAACAAGAAAGCTGGGTATATCACTCTCCTGTGCC
