# Supplementary material for: Evolution, Expression Differentiation and Interaction Specificity of Heterotrimeric G-Protein Subunit Gene Family in the Mesohexaploid Brassica rapa
Source: PLoS One. 2014 Sep 5;9(9):e105771. doi: 10.1371/journal.pone.0105771 (PMC4156303; doi:10.1371/journal.pone.0105771)
Supplement: Table S2 — Amino acid sequence identity (%) of B. rapa Gα, Gβ and Gγ proteins with corresponding proteins from Arabidopsis thaliana ( At ) and rice ( Os ). (PDF) [file pone.0105771.s007.pdf]

Supplementary Table S2. Amino acid sequence identity (%) of *B. rapa* Gα, Gβ and Gγ proteins with corresponding proteins from *Arabidopsis thaliana* (*At*) and rice (*Os*).

	AtGPA1	OsRGA1	BraA.Gα1
AtGPA1	***	66.4	96.9
OsRGA1		***	73.5
BraA.Gα1			***

	AtAGB1	OsRGB1	BraA.Gβ1	BraA.Gβ2	BraA.Gβ3
AtAGB1	***	77.0	89.9	92.9	91.0
OsRGB1		***	73.2	75.0	73.0
BraA.Gβ1			***	90.8	88.7
BraA.Gβ2				***	91.8
BraA.Gβ3					***

	AtAGG1	BraA.Gγ1	AtAGG2	BraA.Gγ2	BraA.Gγ3	AtAGG3	BraA.Gγ4	BraA.Gγ5
AtAGG1	***	71.7	46.5	48.5	42.4	30.3	32.3	31.3
BraA.Gγ1		***	53.3	56.7	51.1	30.0	30.0	31.1
AtAGG2			***	92.1	88.1	37.6	34.7	36.6
BraA.Gγ2				***	86.3	37.3	33.3	36.3
BraA.Gγ3					***	36.6	33.7	35.6
AtAGG3						***	76.6	77.4
BraA.Gγ4							***	71.4
BraA.Gγ5								***
