# Supplementary material for: Evolution, Expression Differentiation and Interaction Specificity of Heterotrimeric G-Protein Subunit Gene Family in the Mesohexaploid Brassica rapa
Source: PLoS One. 2014 Sep 5;9(9):e105771. doi: 10.1371/journal.pone.0105771 (PMC4156303; doi:10.1371/journal.pone.0105771)
Supplement: Table S3 — Syntenic flanking genes of BraA.Gα1 (Bra007761) present in the chromosomal block ‘I’ of B. rapa , as available in BRAD database ( http://www.brassicadb.org/ ). (PDF) [file pone.0105771.s008.pdf]

Supplementary Table S3. Syntenic flanking genes of *BraA.Gα1* (Bra007761) present in the chromosomal block I of *B. rapa*, as available in BRAD database (http://www.brassicadb.org/). The information of *B. rapa* syntenic orthologs of 34 representative *A. thaliana* genes flanking the *AtGPA1* gene is shown. LF, MF1 and MF2 represents the least gene fractionized, moderately gene fractionized and most gene fractionized subgenomes of *B. rapa*, respectively and the linkage group is marked with parenthesis.

**S No.**	***A. thaliana***	***B. rapa***		
		**LF (A09)**	**MF1 (A04)**	**MF2(A03)**
1	At2g26130		Bra034292	
2	At2g26140	Bra007768	Bra034293	
3	At2g26150			Bra000557
4	At2g26160			
5	At2g26170			
6	At2g26180		Bra034294	
7	At2g26190		Bra034295	
8	At2g26200		Bra034296	
9	At2g26210		Bra034297	
10	At2g26230			
11	At2g26240	Bra007767		
12	At2g26250	Bra007766	Bra034298	
13	At2g26260	Bra007764		
14	At2g26267			
15	At2g26270			
16	At2g26280	Bra007762	Bra034300	
17	At2g26290	Bra007786	Bra034302	
**18**	**At2g26300**	**Bra007761**		
19	At2g26310		Bra034304	
20	At2g26320			
21	At2g26330	Bra007759		Bra000555
22	At2g26340	Bra007758		
23	At2g26350	Bra007757		
24	At2g26360		Bra034305	
25	At2g26370			
26	At2g26380			
27	At2g26390			
28	At2g26400			
29	At2g26410			
30	At2g26420	Bra007756		
31	At2g26420			
32	At2g26440			Bra000540
33	At2g26460	Bra007752		
34	At2g26470	Bra007753		
