# Supplementary material for: Evolution, Expression Differentiation and Interaction Specificity of Heterotrimeric G-Protein Subunit Gene Family in the Mesohexaploid Brassica rapa
Source: PLoS One. 2014 Sep 5;9(9):e105771. doi: 10.1371/journal.pone.0105771 (PMC4156303; doi:10.1371/journal.pone.0105771)
Supplement: Table S4 — Relative fold expression of the G-protein genes under various phytohormones and stress treatments at different time points. (PDF) [file pone.0105771.s009.pdf]

Supplementary Table S4. Relative fold expression of the G-protein genes under various phytohormones and stress treatments at different time points. The expression of un-treated seedling was set at 1.

	BraA.Gα1		***BraA.Gβ1***		***BraA.Gβ2***		***BraA.Gβ3***	
	**3hr**	**6hr**	**3hr**	**6hr**	**3hr**	**6hr**	**3hr**	**6hr**
**IAA**	0.43	1.25	0.54	0.97	1.51	8.24	0.86	0.68
**ABA**	0.78	1.03	1.09	0.97	1.80	4.37	0.96	0.80
**SA**	0.78	1.25	0.78	0.66	1.59	4.32	1.04	0.65
**MeJa**	0.74	2.18	0.53	1.02	1.50	12.86	0.75	0.75
**Heat**	0.91	1.08	1.60	1.68	7.90	11.23	1.30	1.01
**Cold**	2.80	4.65	2.09	3.36	9.61	13.91	1.15	2.14
**NaCl**	2.61	1.36	3.07	1.39	6.08	9.40	1.68	0.80

	BraA.Gγ1		***BraA.Gγ2***		***BraA.Gγ3***		***BraA.Gγ4***		***BraA.Gγ5***	
	**3hr**	**6hr**	**3hr**	**6hr**	**3hr**	**6hr**	**3hr**	**6hr**	**3hr**	**6hr**
**IAA**	0.51	0.94	0.73	0.82	0.47	0.77	0.73	0.99	0.23	0.90
**ABA**	0.60	0.69	0.60	0.94	0.59	0.80	0.95	1.06	0.69	0.70
**SA**	0.88	0.93	0.59	0.82	0.55	0.45	0.75	0.79	0.29	4.95
**MeJa**	0.34	0.81	0.57	1.84	0.40	2.11	0.71	1.32	0.75	2.15
**Heat**	0.83	1.14	0.89	1.80	0.75	1.17	1.73	2.01	1.47	8.38
**Cold**	1.16	1.34	2.13	5.81	1.89	1.90	1.68	1.79	0.75	3.08
**NaCl**	1.10	0.35	10.13	1.62	1.57	1.16	2.05	2.30	0.96	0.56
