# Supplementary material for: Evolution, Expression Differentiation and Interaction Specificity of Heterotrimeric G-Protein Subunit Gene Family in the Mesohexaploid Brassica rapa
Source: PLoS One. 2014 Sep 5;9(9):e105771. doi: 10.1371/journal.pone.0105771 (PMC4156303; doi:10.1371/journal.pone.0105771)
Supplement: Table S5 — Nucleotide sequence identity (in %) of 5′ upstream region (1.5 kb of ATG) of B. rapa G-protein genes with corresponding Arabidopsis G-protein promoters. (PDF) [file pone.0105771.s010.pdf]

Supplementary Table S5. Nucleotide sequence identity (in %) of 5’ upstream region (1.5 kb of ATG) of *B. rapa* G-protein genes with corresponding *Arabidopsis* G-protein promoters.

	AtGPA1	*BraA.Gα1*
AtGPA1	-	48.1
BraA.Gα1		-

	AtAGB1	*BraA.Gβ1*	*BraA.Gβ2*	*BraA.Gβ3*
AtAGB1	-	32.9	35.8	33.0
BraA.Gβ1		-	29.7	26.1
BraA.Gβ2			-	28.0
BraA.Gβ3				-

	AtAGG1	*BraA.Gγ1*	*AtAGG2*	*BraA.Gγ2*	*BraA.Gγ3*	*AtAGG3*	*BraA.Gγ4*	*BraA.Gγ5*
AtAGG1	-	41.8	26.3	26.6	28.0	27.9	29.0	29.2
BraA.Gγ1		-	29.7	27.7	29.1	28.6	27.8	27.1
AtAGG2			-	44.2	44.3	31.9	31.7	32.3
BraA.Gγ2				-	48.9	29.6	28.8	29.7
BraA.Gγ3					-	29.8	30.7	33.1
AtAGG3						-	49.8	43.9
BraA.Gγ4							-	42.9
BraA.Gγ5								-
