# Supplementary material for: Evolution, Expression Differentiation and Interaction Specificity of Heterotrimeric G-Protein Subunit Gene Family in the Mesohexaploid Brassica rapa
Source: PLoS One. 2014 Sep 5;9(9):e105771. doi: 10.1371/journal.pone.0105771 (PMC4156303; doi:10.1371/journal.pone.0105771)
Supplement: Table S6 — Summary of cis -regulatory elements present in the 5′ upstream region of the G-protein genes of B. rapa . (PDF) [file pone.0105771.s011.pdf]

Supplementary Table S6. Summary of *cis*-regulatory elements present in the 5' upstream region of the G-protein genes of *B.rapa*. Around 1.5kb region (5' upstream to translation start site ATG) of *B. rapa* G-protein gene was obtained from BRAD database (http://www.brassicadb.org/) and queried in Plant *cis*-acting regulatory DNA elements database (PLACE) (http://www.dna.affrc.go.jp/PLACE/).

**Cis-regulatory elements**	***BraA.Gα1***	***BraA.Gβ1***	***BraA.Gβ2***	***BraA.Gβ3***	***BraA.Gγ1***	***BraA.Gγ2***	***BraA.Gγ3***	***BraA.Gγ4***	***BraA.Gγ5***
-10PEHVPSBD	1	2	0	2	1	3	4	0	0
2SSEEDPROTBANAPA	1	0	0	0	0	0	0	0	0
-300CORE	0	1	0	0	0	1	1	0	0
-300ELEMENT	0	2	1	1	1	3	2	1	1
-300MOTIFZMZEIN	0	0	0	0	0	1	0	0	0
AACACOREOSGLUB1	1	0	1	0	0	0	0	0	0
ABREATCONSENSUS	0	0	0	0	1	0	1	0	0
ABREATRD22	0	0	0	0	1	0	0	0	0
ABRELATERD1	1	3	0	1	2	2	3	2	3
ABRERATCAL	1	1	0	0	2	4	2	1	2
ACGTABOX	0	0	0	0	0	0	0	0	2
ACGTABREMOTIFA2OSEM	0	0	0	0	2	0	1	0	0
ACGTATERD1	6	6	8	4	4	12	4	8	8
ACGTCBOX	0	2	2	0	0	0	0	0	0
ACGTOSGLUB1	0	0	0	0	0	1	0	0	2
ACGTTBOX	2	0	2	2	0	6	0	0	0
ACIIIPVPAL2	1	0	0	0	0	0	0	0	0
AGMOTIFNTMYB2	0	0	0	0	0	0	0	0	1
AMMORESIIUDCRNIA1	0	0	0	0	0	0	0	1	0
AMMORESIVDCRNIA1	0	0	1	0	0	0	0	0	0
AMYBOX1	2	1	1	3	1	1	0	0	0
AMYBOX2	0	0	0	1	0	0	0	0	1
ANAERO1CONSENSUS	6	2	3	0	1	0	1	1	2
ANAERO2CONSENSUS	1	2	0	0	1	0	0	0	0
ARFAT	1	0	4	1	1	2	3	3	1
ARR1AT	21	22	11	16	19	11	13	16	16
ASF1MOTIFCAMV	0	1	1	0	3	1	1	0	2
BIHD1OS	1	2	7	4	2	2	2	5	1
BOXCPSAS1	0	0	2	0	0	0	0	0	0
BOXIINTPATPB	1	3	3	1	1	2	2	1	0
BOXIIPCCHS	0	0	0	0	1	0	1	0	0
BOXLCOREDCPAL	0	0	0	0	1	0	0	1	1
BS1EGCCR	0	1	1	0	1	0	0	0	1
CAATBOX1	15	17	19	11	15	9	20	12	10
CACGTGMOTIF	0	2	0	0	2	0	2	0	0
CACTFTPPCA1	16	19	18	21	21	22	25	16	22
CANBNNAPA	1	0	0	0	1	0	0	0	1
CAREOSREP1	0	0	1	1	0	3	3	0	2
CARGATCONSENSUS	0	0	0	0	0	0	0	2	2
CARGCW8GAT	2	6	4	4	0	0	4	0	2
CATATGGMSAUR	0	0	4	4	0	0	0	2	0
CBFHV	0	2	0	1	0	1	0	1	1
CCA1ATLHCB1	3	0	0	2	1	0	0	0	1
CCAATBOX1	2	6	4	3	4	5	7	5	1
CELLCYCLESC	0	0	1	0	0	0	0	0	0
CEREGLUBOX1PSLEGA	0	0	0	0	0	1	0	0	0
CEREGLUBOX2PSLEGA	0	0	0	1	0	0	0	0	0
CGACGOSAMY3	1	5	3	1	0	7	1	1	4
CGCGBOXAT	6	0	2	0	4	4	4	0	4
CIACADIANLELHC	0	1	3	0	2	0	2	2	0
CMSRE1IBSPOA	0	0	0	0	0	1	0	0	0
CPBCSPOR	0	1	2	0	2	2	1	1	0
CRTDREHVCBF2	0	2	0	0	0	0	0	0	0
CTRMCAMV35S	0	0	0	2	2	0	0	2	2
CURECORECR	8	4	4	2	2	14	6	6	16
DOFCOREZM	34	26	21	19	19	31	42	33	16
DPBFCOREDCDC3	0	2	1	0	3	2	1	1	4
DRE2COREZMRAB17	0	0	0	1	0	0	0	1	1
DRECRTCOREAT	0	0	0	1	0	0	0	1	1
E2F1OSPCNA	0	1	0	0	0	0	0	0	0
E2FANTRNR	0	1	0	0	0	0	0	0	0
E2FCONSENSUS	0	2	1	0	2	0	0	1	1

Cis-regulatory elements	*BraA.Gα1*	*BraA.Gβ1*	*BraA.Gβ2*	*BraA.Gβ3*	*BraA.Gγ1*	*BraA.Gγ2*	*BraA.Gγ3*	*BraA.Gγ4*	*BraA.Gγ5*
EBOXBNNAPA	2	20	20	16	10	8	12	12	6
EECCRCAH1	2	0	0	6	3	2	3	4	0
ELRECOREPCRP1	3	0	0	0	1	1	4	2	0
EMBP1TAEM	0	0	0	0	1	0	1	0	0
ERELEE4	0	0	0	0	1	0	1	0	1
GADOWNAT	0	0	0	0	1	0	0	0	0
GARE1OSREP1	2	0	0	0	0	0	0	0	0
GAREAT	0	1	1	3	1	2	0	1	0
GATABOX	15	12	3	12	7	8	10	16	9
GBOXLERBCS	0	0	0	0	1	0	0	0	0
GCCCORE	0	0	0	0	2	0	0	0	2
GCN4OSGLUB1	0	0	0	0	0	1	0	0	0
GLMHVCHORD	0	0	0	0	0	2	0	0	0
GT1CONSENSUS	18	10	7	16	7	14	15	15	10
GT1CORE	1	0	0	0	0	0	0	0	0
GT1GMSCAM4	6	4	4	4	2	4	8	4	3
GTGANTG10	5	12	10	11	8	10	7	15	6
HDZIP2ATATHB2	0	1	0	1	0	0	0	0	0
HEXAMERATH4	0	2	1	1	0	0	1	0	1
HEXMOTIFTAH3H4	0	0	1	0	0	0	0	0	0
IBOX	2	1	0	0	0	2	0	2	1
IBOXCORE	8	5	2	2	3	6	4	9	5
IBOXCORENT	2	1	0	0	0	2	0	2	1
INRNTPSADB	2	0	1	5	0	1	1	4	1
INTRONLOWER	0	1	0	0	0	0	0	0	0
INTRONUPPER	0	0	0	0	0	0	0	1	0
IRO2OS	0	1	0	0	1	0	1	0	0
L1BOXATPDF1	0	0	1	0	0	0	1	1	0
LEAFYATAG	0	0	0	0	0	0	1	0	0
LECPLEACS2	2	0	0	0	0	0	0	1	1
LRENPCABE	0	0	0	0	1	0	1	0	0
LTRE1HVBLT49	4	0	0	2	1	0	0	0	0
LTRECOREATCOR15	1	0	1	1	1	2	0	1	2
MARARS	2	0	0	0	0	0	0	0	0
MARTBOX	14	2	2	7	0	2	0	2	2
MYB1AT	2	2	5	4	1	3	2	2	2
MYB1LEPR	0	0	1	0	0	1	0	0	1
MYB26PS	1	0	0	0	0	0	0	0	0
MYB2AT	0	3	0	1	0	0	0	0	2
MYB2CONSENSUSAT	1	6	2	3	2	3	1	3	4
MYBCORE	6	8	4	6	5	3	2	4	6
MYBCOREATCYCB1	1	2	1	2	3	2	1	3	2
MYBGAHV	0	1	1	3	1	1	0	0	0
MYBPLANT	1	0	0	0	1	0	0	0	0
MYBPZM	1	0	0	0	3	3	2	3	0
MYBST1	4	2	1	3	2	1	1	2	2
MYCATERD1	0	3	0	2	0	0	2	1	1
MYCATRD22	0	3	0	2	0	0	2	1	1
MYCCONSENSUSAT	2	20	20	16	10	8	12	12	6
NAPINMOTIFBN	0	1	0	0	1	0	0	0	1
NODCON1GM	4	1	0	4	1	3	2	5	2
NODCON2GM	4	6	8	4	5	8	9	6	6
NTBBF1ARROLB	0	0	1	0	0	3	1	1	1
O2F3BE2S1	0	0	0	0	0	1	0	0	0
OSE1ROOTNODULE	4	1	0	4	1	3	2	5	2
OSE2ROOTNODULE	4	6	8	4	5	8	9	6	6
P1BS	0	0	2	0	0	0	0	0	2
PALBOXAPC	0	0	0	0	1	1	0	0	0
POLASIG1	6	4	2	1	2	4	2	4	5
POLASIG2	0	1	0	2	1	2	0	0	1
POLASIG3	5	4	2	4	0	2	2	0	1
POLLEN1LELAT52	14	5	11	9	2	12	15	18	12
PREATPRODH	1	2	1	3	1	2	1	2	0
PRECONSCRHSP70A	0	1	2	2	2	3	1	3	4
PROLAMINBOXOSGLUB1	0	0	0	1	0	0	1	1	1
PYRIMIDINEBOXHVEPB1	0	0	0	0	0	0	1	1	0
PYRIMIDINEBOXOSRAMY1A	4	3	1	0	4	1	2	2	1
QARBNEXTA	1	0	0	0	0	1	0	0	0
QELEMENTZMZM13	1	0	1	1	1	0	1	0	0
RAV1AAT	5	3	10	15	3	4	5	2	4

**Cis-regulatory elements**	***BraA.Gα1***	***BraA.Gβ1***	***BraA.Gβ2***	***BraA.Gβ3***	***BraA.Gγ1***	***BraA.Gγ2***	***BraA.Gγ3***	***BraA.Gγ4***	***BraA.Gγ5***
RAV1BAT	0	1	1	0	1	0	0	1	0
RBCSCONSENSUS	0	0	1	0	0	0	2	0	0
REALPHALGLHCB21	3	3	2	4	3	2	2	0	0
REBETALGLHCB21	3	1	0	0	0	0	0	0	0
RHERPATEXPA7	0	3	1	0	2	0	1	2	0
ROOTMOTIFTAPOX1	20	9	8	8	4	4	9	10	9
RYREPEATBNNAPA	0	1	1	1	0	0	0	1	2
RYREPEATGMGY2	0	0	1	0	0	0	0	0	0
RYREPEATLEGUMINBOX	0	0	1	0	0	0	0	0	0
S1FBOXSORPS1L21	1	0	0	0	0	5	2	3	1
SBOXATRBCS	0	0	0	1	0	0	0	0	0
SEBFCONSSTPR10A	0	0	3	1	0	1	2	4	0
SEF1MOTIF	0	0	0	0	1	0	0	0	0
SEF3MOTIFGM	1	0	1	1	1	2	2	2	0
SEF4MOTIFGM7S	1	4	3	3	1	1	2	5	3
SITEIIATCYTC	2	1	0	0	5	3	3	2	2
SITEIOSPCNA	0	0	0	0	0	0	0	1	0
SORLIP1AT	0	2	0	1	2	3	2	1	2
SORLIP2AT	1	1	0	0	4	2	2	2	2
SORLIP5AT	0	0	2	0	0	0	0	0	0
SORLREP3AT	0	0	0	0	0	0	0	1	1
SP8BFIBSP8BIB	0	0	0	0	2	2	0	1	0
SREATMSD	2	1	1	1	0	0	0	1	1
SURE1STPAT21	0	0	1	0	0	0	0	0	0
SURECOREATSULTR11	1	2	6	4	4	4	6	5	3
SV40COREENHAN	0	0	0	0	0	2	1	0	0
T/GBOXATPIN2	1	0	0	0	0	1	0	1	0
TAAAGSTKST1	4	4	3	1	4	9	7	3	4
TATABOX2	0	1	2	0	0	0	0	1	1
TATABOX3	0	0	0	1	0	0	0	0	0
TATABOX4	2	0	1	2	1	2	3	5	1
TATABOX5	9	5	1	2	1	1	1	2	5
TATABOXOSPAL	2	0	1	0	1	2	3	0	0
TATAPVTRNALEU	1	0	1	1	0	1	1	2	0
TATCCACHVAL21	0	0	0	0	0	0	1	0	0
TATCCAOSAMY	1	0	0	1	1	0	1	1	1
TATCCAYMOTIFOSRAMY3D	0	0	0	1	0	0	1	0	1
TBOXATGAPB	1	2	0	0	1	0	2	3	0
TE2F2NTPCNA	0	0	1	0	1	0	0	0	0
TGACGTVMAMY	0	0	1	0	0	0	0	0	0
TGTCACACMCUCUMISIN	0	0	0	1	0	0	0	1	0
TRANSINITDICOTS	0	0	0	0	0	0	1	0	0
TRANSINITMONOCOTS	0	0	0	0	0	0	1	1	0
UP1ATMSD	0	1	0	0	1	1	1	0	1
UP2ATMSD	0	1	0	0	0	0	0	0	0
UPRMOTIFIIAT	0	0	0	0	1	0	2	0	0
WBBOXPCWRKY1	1	0	0	3	0	2	3	0	0
WBOXATNPR1	3	4	5	8	3	5	4	5	1
WBOXHVISO1	1	3	2	8	2	6	4	2	2
WBOXNTCHN48	0	0	1	1	0	0	1	0	1
WBOXNTERF3	4	3	3	9	3	7	9	5	3
WRKY71OS	5	6	11	13	8	10	12	10	6
WUSATAg	0	0	0	0	0	0	0	0	1
XYLAT	1	1	0	0	0	1	1	2	0
