# Supplementary material for: Evolution, Expression Differentiation and Interaction Specificity of Heterotrimeric G-Protein Subunit Gene Family in the Mesohexaploid Brassica rapa
Source: PLoS One. 2014 Sep 5;9(9):e105771. doi: 10.1371/journal.pone.0105771 (PMC4156303; doi:10.1371/journal.pone.0105771)
Supplement: Figure S1 — Nucleotide sequence alignment of the reported (Bra007761) and amplified BraA.Gα1 coding DNA sequences along with their translated amino-acid sequences. (PDF) [file pone.0105771.s001.pdf]

```
                                                                                                                          G1 Domain I2                                I3
1    M G L L C S R S R H H T E D T D E N A Q A A E I E R R I E Q E A K A E K H I R K L L L L G A K - - - F W V L T L Q I K L   Bra007761
1    ATGGGCTTACTCTGCAGTAGAAGTCGACATCATACAGAAGATACCGACGAGAATGCACAGGCTGCTGAAATCGAAAGACGGATAGAGCAAGAGGCAAAGGCTGAAAAACATATTCGGAAACTTCTCCTACTTGGAGCAAAG---------TTTTGGGTGCTAACTTTGCAGATAAAACTT
1    M G L L C S R S R H H T E D T D E N A Q A A E I E R R I E Q E A K A E K H I R K L L L L G A G E S G K S T I F K Q I K L   BraA.GαR1
1    ATGGGCTTACTCTGCAGTAGAAGTCGACATCATACAGAAGATACCGACGAGAATGCACAGGCTGCTGAAATCGAAAGACGGATAGAGCAAGAGGCAAAGGCTGAAAAACATATTCGGAAACTTCTCCTACTTGGTGCTGGAGAATCAGGAAAGTCTACTATTTTTAAGCAGATAAAACTT

58   L F Q T G F D E G E L K S Y V P V I H A N V Y Q T I K L L H D G T K E F A Q N E T D P A K Y T L S S E N M A I G E K L S   Bra007761
172  CTCTTCCAAACTGGATTCGATGAAGGAGAGCTAAAGAGCTATGTTCCGGTCATCCATGCCAATGTCTATCAGACTATAAAATTATTGCATGATGGAACGAAGGAGTTTGCTCAAAATGAAACTGATCCTGCAAAATATACGTTATCCTCTGAAAATATGGCCATTGGGGAGAAACTATCT
61   L F Q T G F D E G E L K S Y V P V I H A N V Y Q T I K L L H D G T K E F A Q N E T D P A K Y T L S S E N M A I G E K L S   BraA.GαR1
181  CTCTTCCAAACTGGATTCGATGAAGGAGAGCTAAAGAGCTATGTTCCGGTCATCCATGCCAATGTCTATCAGACTATAAAATTATTGCATGACGGAACGAAGGAGTTTGCTCAAAATGAAACTGATCCTGCAAAATATACGTTATCCTCTGAAAATATGGCCATTGGGGAGAAACTATCT

118  E I G A R L D Y P R L T K D L A E G I E T L W N D P A I Q E T C S R G N E L Q V P D C T K Y L M E N L K R L S D V N Y I   Bra007761
352  GAAATTGGAGCTAGGTTAGACTATCCGCGTCTTACCAAGGACCTCGCTGAGGGAATAGAAACACTATGGAATGATCCTGCTATCCAGGAAACATGTTCTCGTGGTAATGAGCTTCAGGTTCCCGATTGTACTAAATATCTGATGGAGAACTTGAAGAGACTTTCAGATGTCAACTATATT
121  E I G A R L D Y P R L T K D L A E G I E T L W N D P A I Q E T C S R G N E L Q V P D C T K Y L M E N L K R L S D V N Y I   BraA.GαR1
361  GAAATTGGAGCTAGGTTAGACTATCCGCGTCTTACCAAGGACCTCGCCGAGGGAATAGAAACACTATGGAATGATCCTGCTATCCAGGAAACATGTTCTCGTGGTAATGAGCTTCAAGTTCCCGATTGTACTAAATATCTGATGGAGAATTTGAAGAGATTATCAGATGTCAACTATATT

178  P T K E D V L Y A R V R T T G V V E I Q F S P V G E N K K S G E V Y R L F D V G G Q R N E R R K W I H L F E G V T A V I   Bra007761
532  CCAACCAAGGAGGATGTTCTGTATGCGAGAGTTCGCACAACTGGTGTCGTGGAAATACAGTTCAGCCCTGTGGGAGAAAATAAGAAAAGTGGTGAAGTGTACCGATTGTTTGACGTGGGTGGACAGAGAAATGAGAGAAGGAAATGGATTCATCTGTTTGAAGGTGTTACTGCTGTTATT
181  P T K E D V L Y A R V R T T G V V E I Q F S P V G E N K K S G E V Y R L F D V G G Q R N E R R K W I H L F E G V T A V I   BraA.GαR1
541  CCAACCAAGGAGGATGTTCTGTATGCGAGAGTTCGCACAACTGGTGTCGTGGAAATACAGTTCAGCCCCGTGGGAGAAAATAAGAAAAGTGGTGAAGTGTACCGATTGTTTGACGTGGGTGGACAGAGAAATGAGAGAAGGAAATGGATTCATCTGTTTGAAGGTGTTACTGCTGTTATT

238  F C A A I S E Y D Q T L F E D E Q K N R M M E T K E L F D W V L K Q P C F E K T S I M L F L N K F D I F E K K V L D V P   Bra007761
712  TTCTGTGCTGCCATCAGCGAGTACGACCAAACACTATTTGAGGACGAGCAGAAAAACAGGATGATGGAGACCAAAGAACTGTTCGACTGGGTCTTGAAACAGCCCTGTTTCGAGAAAACATCCATCATGTTATTCCTCAACAAGTTTGACATATTTGAGAAGAAGGTTCTTGATGTTCCG
241  F C A A I S E Y D Q T L F E D E Q K N R M M E T K E L F D W V L K Q P C F E K T S I M L F L N K F D I F E K K V L D V P   BraA.GαR1
721  TTCTGTGCTGCCATCAGCGAGTACGACCAAACACTATTTGAGGACGAGCAGAAAAACAGGATGATGGAGACCAAAGAGCTGTTCGACTGGGTCTTGAAACAGCCCTGTTTCGAGAAAACATCCATCATGCTATTCCTCAACAAGTTTGACATATTTGAGAAGAAGGTTCTTGATGTTCCG

298  L N V C E W F R D Y Q P V S S G K Q E I E H A Y E F V K K K F E E L Y Y Q N T A P D R V D R V F K I Y R T T A L D Q K L   Bra007761
892  TTGAATGTTTGCGAGTGGTTCAGAGATTACCAACCAGTTTCGAGTGGGAAACAAGAGATCGAGCATGCCTACGAGTTTGTGAAGAAGAAATTTGAGGAATTATATTACCAGAACACTGCGCCGGATCGAGTGGACAGGGTGTTTAAGATCTACAGGACGACGGCTTTGGATCAGAAGCTT
301  L N V C E W F R D Y Q P V S S G K Q E I E H A Y E F V K K K F E E L Y Y Q N T A P D R V D R V F K I Y R T T A L D Q K L   BraA.GαR1
901  TTGAATGTTTGCGAGTGGTTCAGAGATTACCAACCAGTTTCGAGTGGGAAACAAGAGATCGAGCATGCCTACGAGTTTGTGAAGAAGAAATTTGAGGAATTATATTACCAGAACACTGCGCCGGATCGAGTGGACAGGGTGTTTAAGATCTACAGGACGACGGCTTTGGATCAGAAGCTT

358  V K K T F K L V D E T L R R R N L L E A G L L .   Bra007761
1072 GTGAAGAAAACGTTCAAGCTTGTAGATGAGACTCTAAGAAGGAGAAACCTCTTGGAGGCTGGTCTTTTATGA
361  V K K T F K L V D E T L R R R N L L E A G L L .   BraA.GαR1
1081 GTGAAGAAAACGTTCAAGCTTGTAGATGAGACTCTAAGAAGGAGAAACCTCTTGGAGGCTGGCCTTTTATGA
```

Supplementary Figure S1. Nucleotide sequence alignment of the reported (Bra007761) and amplified *BraA.Gα1* coding DNA sequences along with their translated amino-acid sequence. Positions of second and third introns spanning the mis-annotated G1 domain are marked.
