# Supplementary material for: Evolution, Expression Differentiation and Interaction Specificity of Heterotrimeric G-Protein Subunit Gene Family in the Mesohexaploid Brassica rapa
Source: PLoS One. 2014 Sep 5;9(9):e105771. doi: 10.1371/journal.pone.0105771 (PMC4156303; doi:10.1371/journal.pone.0105771)
Supplement: Figure S2 — Nucleotide sequence alignment of 5′ upstream regions of BraA.Gα1 (Bra007761) with AtGPA1 . (PDF) [file pone.0105771.s002.pdf]

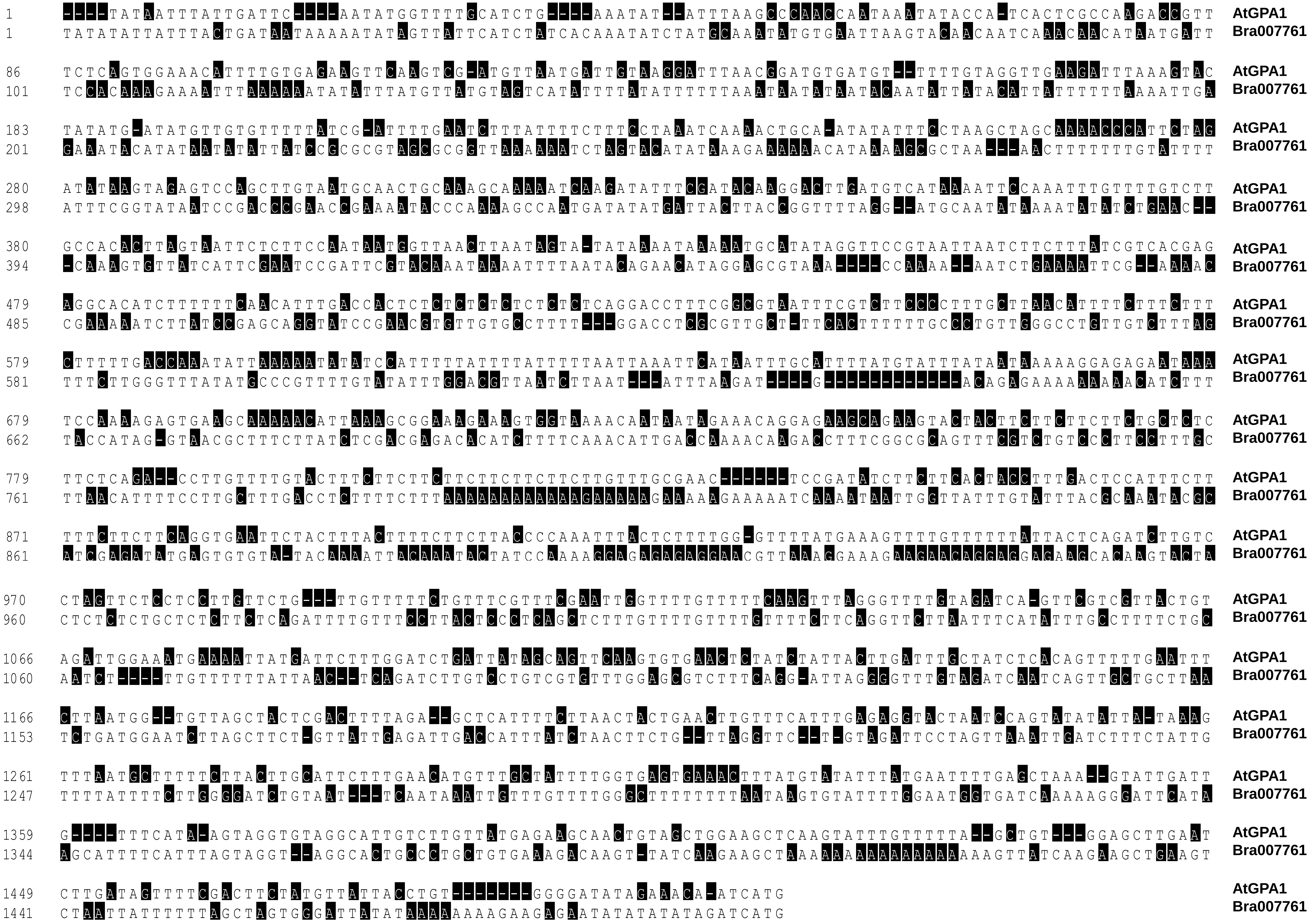

Supplementary Figure S2. Nucleotide sequence alignment of 5' upstream regions of *BraA.Gα1* (Bra007761) with *AtGPA1*. Sequence alignment of 1.5 kb sequence upstream to putative translation start site (ATG) was performed using Clustal W. The residues differing from consensus are marked in dark background.
