# Supplementary material for: Evolution, Expression Differentiation and Interaction Specificity of Heterotrimeric G-Protein Subunit Gene Family in the Mesohexaploid Brassica rapa
Source: PLoS One. 2014 Sep 5;9(9):e105771. doi: 10.1371/journal.pone.0105771 (PMC4156303; doi:10.1371/journal.pone.0105771)
Supplement: Figure S4 — Nucleotide sequence alignment of 5′ upstream regions of BraGγ genes. (PDF) [file pone.0105771.s004.pdf]

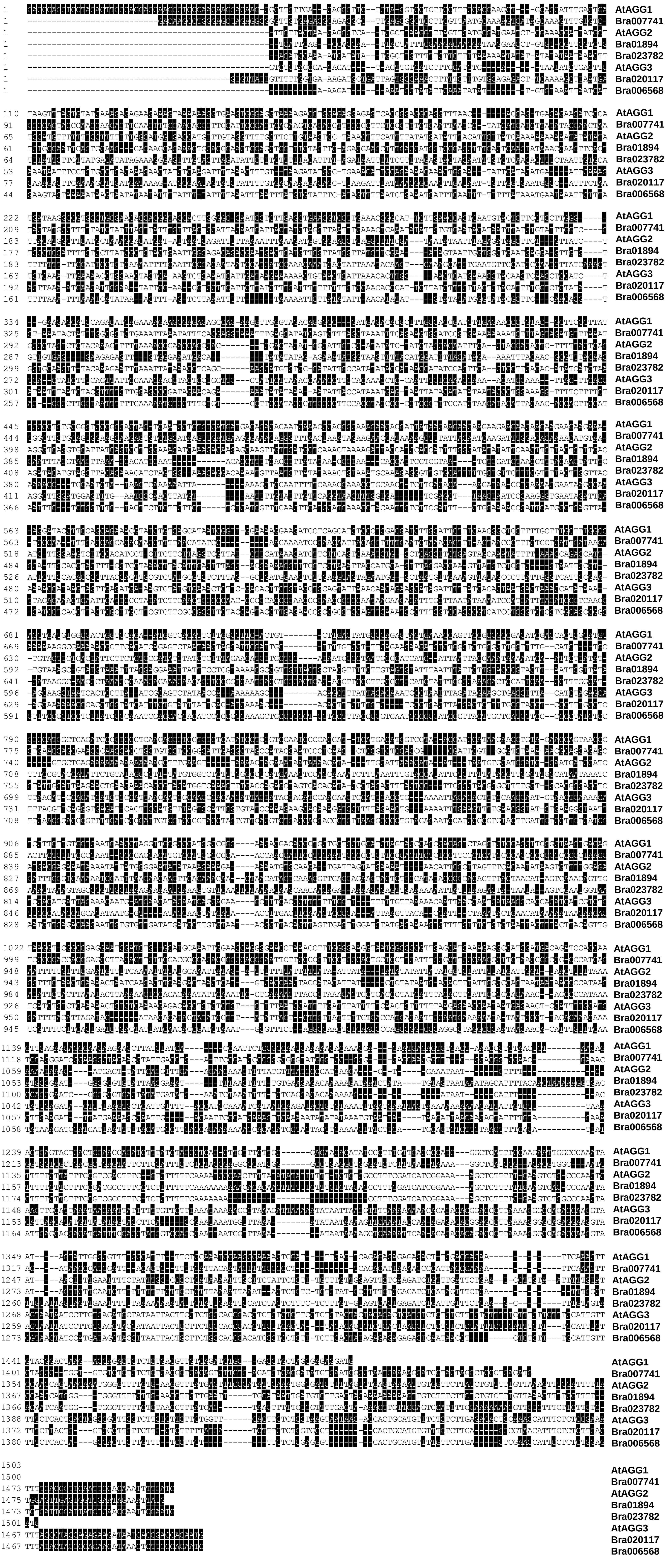

Supplementary Figure S4. Nucleotide sequence alignment of 5' upstream regions of *BraGγ* genes. Nucleotide sequence alignment of 1.5 kb sequence upstream to putative translation start site (ATG) of *BraA.Gγ1* (Bra007741), *BraA.Gγ2* (Bra001894), *BraA.Gγ3* (Bra023782), *BraA.Gγ4* (Bra020117), *BraA.Gγ5* (Bra006568), and Arabidopsis *Gγ* genes (*AtAGG1*, *AtAGG2* and *AtAGG3*) was performed using Clustal W. The residues differing from consensus are marked in dark background.
